# From Genotype × Environment Interaction to Gene × Environment Interaction 

**DOI:** 10.2174/138920212800543066

**Published:** 2012-05

**Authors:** Jose Crossa

**Affiliations:** Biometrics and Statistics Unit, International Maize and Wheat Improvement Center (CIMMYT), Apdo. Postal 6-641, 06600 Mexico, D.F., Mexico

**Keywords:** Genotype × environment interaction (GE), gene × environment interaction, environmental and genotypic covariables, Quantitative Trait Loci (QTL), molecular markers (MM), Genomics-enable prediction and selection.

## Abstract

Historically in plant breeding a large number of statistical models has been developed and used for studying genotype × environment interaction. These models have helped plant breeders to assess the stability of economically important traits and to predict the performance of newly developed genotypes evaluated under varying environmental conditions. In the last decade, the use of relatively low numbers of markers has facilitated the mapping of chromosome regions associated with phenotypic variability (e.g., QTL mapping) and, to a lesser extent, revealed the differetial response of these chromosome regions across environments (i.e., QTL × environment interaction). QTL technology has been useful for marker-assisted selection of simple traits; however, it has not been efficient for predicting complex traits affected by a large number of loci. Recently the appearance of cheap, abundant markers has made it possible to saturate the genome with high density markers and use marker information to predict genomic breeding values, thus increasing the precision of genetic value prediction over that achieved with the traditional use of pedigree information. Genomic data also allow assessing chromosome regions through marker effects and studying the pattern of covariablity of marker effects across differential environmental conditions. In this review, we outline the most important models for assessing genotype × environment interaction, QTL × environment interaction, and marker effect (gene) × environment interaction. Since analyzing genetic and genomic data is one of the most challenging statistical problems researchers currently face, different models from different areas of statistical research must be attempted in order to make significant progress in understanding genetic effects and their interaction with environment.

## INTRODUCTION

The presence of genotype × environment interaction (GE) in plant breeding multi-environment trials (MET) is expressed either as inconsistent responses of some genotypes relative to others due to genotypic rank change or as changes in the absolute differences between genotypes without rank change (i.e., heterogeneity of within-site variance). Several models are commonly used for describing the mean response of genotypes across environments and for studying and interpreting GE in agricultural experiments: linear models, bilinear models, and linear-bilinear models. Fixed-effect linear-bilinear models, such as the Sites Regression (SREG) [1, 2] and the Additive Main effect and Multiplicative Interaction (AMMI) models [3, 4] are used for studying genotypic response patterns across environments. In these models, the response patterns of genotypes and environments can be visualized graphically using biplots [5, 6] that allow the breeder to observe the high performing genotype(s) in a region(s) and/or sub-region(s). Recently, several review articles pointed out the merits and demerits of the fixed-effect linear-bilinear models [7-11]. One class of fixed-effect linear models, namely factorial regression (FR) models, and one class of bilinear models, namely partial least squares (PLS) regression, allow incorporating external environmental and genotypic covariables directly into the model and are useful for finding the climatic causes of GE or the genetic factors (molecular markers) influencing GE. 

Linear mixed models have become widely accepted and used for analyzing MET in plant breeding [12-19]. The models naturally lead to a factor analytic (FA) [20, 21] form of the genetic variance-covariance for environments that is more parsimonious and flexible than other variance-covariance structures. Since these are linear-bilinear mixed models, they also have the usual advantages, i.e., that error variance modeling can be accommodated (in particular, heterogeneity of block and error variance between environments and within-environment spatial correlation) and that incomplete data are handled with ease. Furthermore, when genotypes are considered as random effects, coefficients of parentage can be incorporated into the FA model for GE, thereby obtaining more precise estimates of the breeding values of genotypes [16, 22, 18]. Furthermore, Burgueño *et al*. [19] showed how to use the FA model and its biplot for clustering sites and genotypes with statistically negligible crossover interaction (COI). The method proposed by Burgueño *et al*. [19] has two main advantages: (1) the descriptive biplot is the starting point for testing successive hypotheses about the suitability of combining sites and genotypes into subsets that decrease the amount of significant COI, and (2) the process of delineating mega-environments and (3) answering the question “who wins where?” can be done within the framework of linear mixed models. 

As for comparing the predictive ability of linear-bilinear fixed-effect models, the study of Cornelius and Crossa [23] used a cross-validation scheme that splits the data of a MET into a training set and a validation set, and computes the root mean squared predictive error between the predictive value and the observed value of the model. Those authors evaluated the within-trial predictive assessment of several fixed-effect linear-bilinear models, and developed efficient shrinkage estimates of fixed linear-bilinear models that showed the same or better predictive ability than the traditional Best Linear Unbiased Prediction (BLUP) of the cell mean. However, Cornelius and Crossa [23] only studied the predictive ability of models within specific environments (within-trial prediction) but did not assess the prediction of performance of unobserved genotypes in other environments (between-trial prediction). Also, Piepho [21] performed cross-validation on linear-bilinear models but in a slightly different form. Fixed linear-bilinear models have been used for describing GE or the combination of genotype plus GE interaction (GGE) based on a biplot graph. Recently, Burgueño *et al*. [24] compared the predictive ability of various linear-bilinear models and mixed effects models using the factor analytic model; results show that for data sets with complex GE or GGE, modeling GE and GGE using the FA model improved the predictability of the model up to 5-7%. When GE and GGE were not complex, most models gave high predictability (FA versus no FA) and FA did not seem to lose much predictability (only 2%). Therefore, it was concluded that modeling GE and GGE is a good thing.

Selection in plant breeding is usually based on estimates of breeding values obtained with pedigree-based mixed models. In their multivariate formulation, these models can also accommodate genotype × environment (GE) interaction. These models have been used successfully for predicting breeding values in plants and animals. However, pedigree-based models cannot account for Mendelian segregation, a term that, under an infinitesimal additive model (e.g., Fisher [25]) and in the absence of inbreeding, explains one half of the genetic variability. Molecular markers allow tracing Mendelian segregation at several positions of the genome, which gives them enormous potential in terms of increasing the accuracy of estimates of genetic values and the genetic progress attainable when these predictions are used for selection purposes. 

The models mentioned above assess GE from an overall perspective, that is, they do not attempt to decompose or partition the total GE into chromosome regions or even further into specific genes so that the physical genetic causes of GE can be identified in the chromosome. Although Quantitative Trait Loci (QTL) mapping has been routinely used in plant breeding, approaches that fully exploit data from MET to assess and study QTL × environment interaction (QEI) are very limited. The modeling of genetic (co)variances between environments, in combination with modeling of heterogeneous residuals, is an important condition for reaching reliable conclusions about the main effects of QTLs as well as the QEI that is caused by the different expressions of QTLs in different environments; linear mixed models are the natural framework for analyzing such complex data. Multi-environment QTL mapping approaches have been presented in the literature [26]. In these examples, single-trait QTL mapping is studied so that the problem is reduced to either a multi-trait or a multi-environment dimension, but not both. The QTL linear mixed model can be extended to cover both multi-trait and multi-environment cases by first identifying the genetic correlation between traits and/or environments and by imposing some structure on the (co)variance matrix, and then incorporating molecular marker information to extend the phenotypic model into the QTL model [27]. Marker-assisted selection (MAS) and the identification of molecular markers closely linked to QTLs [28] have been widely used in plant breeding to improve a few traits controlled by major genes. However, adoption of the technology has been limited because the biparental populations used for mapping QTLs are not easily used in breeding applications. Also, since MAS uses only partial information (few markers), it presents limitations for improving traits controlled by many loci with small effects because the few markers linked to significant QTLs explain only a small percentage of the total genetic variability (the problem of missing heritability). 

On the other hand, genomic selection (GS) (or genome-wide selection) is an approach for improving quantitative traits [29] that uses all available molecular markers (MMs) across the genome to estimate genetic values. Reports on the use of GS in plants are few and refer mainly to computer simulation studies such as the research of Bernardo and Yu [30], who concluded that GS is superior to marker-assisted selection in maize. In recent articles, de los Campos *et al*. [31], Crossa *et al*. [32, 33], and Perez *et al*. [34] used Bayesian estimates from genomic parametric and semi-parametric regression and showed that models using MMs produced more accurate predictions of grain yield and other traits in maize and wheat than those based only on pedigree. Genomic selection has been validated in animal breeding for predicting breeding values [35, 36, 31].

In a usual genetic model, the phenotypic response of the i^th^ individual (y_i_) is described as the sum of a genetic value, g_i_, and a model residual, ε_i_ , such that the linear model for the genotypes (i=1,2,..,n) is represented as y_i_ = µ + g_i_ + ε_i_ (where µ is the general mean). One method for incorporating markers in models for GS is to define g_i_ as a parametric regression on marker covariates X_ij_ (which can take values of 1, 0, and -1 for a biallelic marker of a segregating population or values of 1 and -1 for inbred lines) of the form $g_{i}=\sum_{j=1}^{p}x_{\mathrm{ij}}\beta_{j}$ , such that $y_{i}=\sum_{j=1}^{p}x_{\mathrm{ij}}\beta_{j}+\varepsilon_{i}$ (j=1,2,…,p), where β_j_ is the regression ofy_i_ on the j^th^ marker covariate. In matrix notation, the model is expressed as y=Xβ+ε . Usually, the number of markers exceeds the number of individuals, and estimation of marker effects *via *ordinary least squares (OLS) is not feasible. In OLS, estimates are obtained to maximize model goodness-of-fit to the training set, and model complexity is not considered. When the number of MMs is large, this typically yields high mean-squared error of estimates of marker effects and poor predictive ability.

In the rest of the chapter I will show the basic fixed and mixed linear-bilinear models underlying GE, the models employed to develop QTL mapping and QTL×E interaction assessment, and the basic models for genomic-enable prediction that allow assessing gene × environment interaction..

## STATISTICAL MODELS FOR ASSESSING GENOTYPE × ENVIRONMENT INTERACTION

### The Basic Model

The basic two-way fixed effects linear model for GE analyses considers that the empirical mean response, ${\bar{y}}_{\mathrm{ij}}$ , of the i^th^ genotype (i=1,2,…,g) in the j^th^ environment (j=1,2,…,s) with r replications in each of the g×s cells is expressed as

(1) $${\bar{y}}_{\mathrm{ij}}=\mu +\tau_{i}+\delta_{j}+{\left(\tau \delta \right)}_{\mathrm{ij}}+{\bar{\varepsilon }}_{\mathrm{ij}}$$

where µ is the grand mean over all genotypes and environments, τ_i_ is the main effect of the i^th^ genotype, δ_j_ is the main effect of the j^th^ environment, (τδ)_ij_ is the effect of the interaction (GE) of the i^th^ genotype in the j^th^ environment, and ${\bar{\varepsilon }}_{\mathrm{ij}}$ is the average error, assumed to be NID ( $0,\sigma_{\varepsilon }^{2}/r$ ) (where $\sigma_{\varepsilon }^{2}$ is the within-environment error variance, assumed to be constant, and r is the number of observations per cell). For a complete random model, it is assumed thatτ_i_, δ_j_, and ( τδ)_ij_ are normally and independently distributed, with variances $\sigma_{\tau }^{2}$ , $\sigma_{\delta }^{2}$ , and $\sigma_{\tau \delta }^{2}$ , respectively. Adding the design effects to (1) with randomized complete blocks, or any type of incomplete block design, does not pose a problema. Furthermore, modeling the residulas by means of spatial analyses does not present further difficulty and is a practice that must be routinely used in any field experiment.

Yates and Cochran [37] introduced a model in which the GE term is linearly related to the environmental main effect ${\left(\tau \delta \right)}_{\mathrm{ij}}$= ϑ_i_ ${\bar{y}}_{\mathrm{ij}}$ , such that the stability parameter б_i_ is the regression of genotype performance on the environment mean ${\bar{y}}_{.j}$ . This was later more formally presented by Finlay and Wilkinson [38] and extended by Eberhart and Russell [39] to include the deviation from regression as another statility parameter (although this is in fact a lack or fit of the model).

### Fixed Effect Linear-Bilinear Models

Williams [40] considered the model ${\bar{y}}_{\mathrm{ij}}=\mu +\tau_{i}+\lambda \alpha_{i}\gamma_{j}+{\bar{\varepsilon }}_{\mathrm{ij}}$ , where λ is the largest singular value of **ZZ*'*** and **Z*'*Z** (for **Z**= ${\bar{y}}_{\mathrm{ij}}-{\bar{y}}_{i.}$ ) and α_i_ and γ_j_ are the corresponding eigenvectors. Gollob [41] and Mandel [42] extended Williams’ [40] work by considering the bilinear GE term as ${\left(\tau \delta \right)}_{\mathrm{ij}}=\sum_{k}^{t}=1\lambda_{k}\alpha_{\mathrm{ik}}\gamma_{\mathrm{jk}}$ . Thus, the general formulation of the linear-bilinear model is

(2) $${\bar{y}}_{\mathrm{ij}}=\mu +\tau_{i}+\delta_{j}+\sum_{k=1}^{t}\lambda_{k}\alpha_{\mathrm{ij}}\gamma_{\mathrm{jk}}+{\bar{\varepsilon }}_{\mathrm{ij}}$$

where the constant λ_k_ is the singular value of the k^th^ bilinear (multiplicative) component that is ordered λ_1_ ≥λ_2_ ≥...≥λ_t_; the α_ik_ are elements of the k^th ^left singular vector of the true interaction and represent genotypic sensitivity to hypothetical environmental factors represented by the k^th^ right singular vector with elements γ_jk_. The α_ik_ and γ_jk_ satisfy the constraints $\sum_{i=1}^{g}\alpha_{\mathrm{ik}}\alpha_{ik^{\prime }}=\sum_{j=1}^{s}\gamma_{\mathrm{jk}}\gamma_{jk^{\prime }}=0$ for k≠k*'* and $\sum_{i}\alpha_{\mathrm{ik}}^{2}=\sum_{j}\gamma_{\mathrm{jk}}^{2}=1$ . Gabriel [43] described the least squares fit of Eq. 2 and explained how the the GE term, **Z**= ${\bar{y}}_{\mathrm{ij}}-{\bar{y}}_{i.}-{\bar{y}}_{.j}+{\bar{y}}_{..}$ , is subjected to singular value decomposition (SVD) after adjusting for the additive (linear) terms. Gauch [3] called Eq. 2 the Additive Main effects and Multiplicative Interaction (AMMI) model.

Other classes of linear-bilinear models described by Cornelius *et al*. [1] are the Genotypes Regression Model (GREG) ${\bar{y}}_{\mathrm{ij}}=\mu_{i}\sum_{k}^{t}=1\lambda_{k}\alpha_{\mathrm{ik}}\gamma_{\mathrm{jk}}+{\bar{\varepsilon }}_{\mathrm{ij}},$ the Sites (environments) Regression Model (SREG) ${\bar{y}}_{\mathrm{ij}}=\mu_{j}\sum_{k}^{t}=1\lambda_{k}\alpha_{\mathrm{ik}}\gamma_{\mathrm{jk}}+{\bar{\varepsilon }}_{\mathrm{ij}},$ the Completely Multiplicative Model ${\bar{y}}_{\mathrm{ij}}=\mu +\sum_{k}^{t}=1\lambda_{k}\alpha_{\mathrm{ik}}\gamma_{\mathrm{jk}}+{\bar{\varepsilon }}_{\mathrm{ij}},$ (COMM), and the Shifted Multiplicative Model (SHMM) ${\bar{y}}_{\mathrm{ij}}=\beta +\sum_{k}^{t}=1\lambda_{k}\alpha_{\mathrm{ik}}\gamma_{\mathrm{jk}}+{\bar{\varepsilon }}_{\mathrm{ij}},$ . 

In matrix notation, these linear-bilinear models can be expressed as **Y**= $\sum_{k=1}^{m}\beta_{k}$ **X**_k_ + **AΛG*'***+**E** [44], where **Y**=[ ${\bar{y}}_{\mathrm{ij}}$ ], **X**_k_=[ $x_{\mathrm{kij}}$ ], **E**=[ ${\bar{\varepsilon }}_{\mathrm{ij}}$ ], **Λ**=diag(λ_k_, k=1,2,…,t), λ_1_ ≥λ_2_ ≥...≥λ_t_, **A**=(**α**_1_,...,**α**_t_), **G**=(γ_1_,...,γ_t_), and **A*'*A**=**G*'*G**=**I**_t_. The x_kij_ are known constants and β_k_, λ_k_, α_ik_, and γ_jk_ are parameters to be estimated. It is possible to study GE by finding low dimensional approximations by means of the singular value decomposition of the structure present in the two-way table.

The GREG linear-bilinear model defined above is a reparameterization of the stability analysis model of Finlay and Wilkinson [38] and the Eberhart and Russell [39] models that perform the linear regressions of genotype on environment means. In the GREG model the first multiplicative term, λ_1_α_i1_γ_j1_is perceived as the genotype regressions, with coefficients α_i1_ on environmental indices γ_j1_ (the scale parameter λ_1_ can be absorbed into α_i1_ or γ_j1_ or partially into each), and the deviation modeled as multiplicative components, provided that *t*>1.

There are several statistical as well as biological reasons to prefer SREG over AMMI for assessing COI and non-COI under the common situation of complex GE: (1) for the same number of bilinear terms, SREG is a more parsimonious model than AMMI; (2) SREG incorporates the main effect of genotypes directly into the statistical analysis of GE, that is, both effects genotypes and GE (GGE) are combined and estimated jointly; this is important for reaching breeders’ objectives, which requires including the main performance of genotypes in the model; (3) sometimes the mixed SREG model can be fitted much more easily than the mixed AMMI model; and (4) the mixed SREG model, as proved by Burgueño *et al*. [19], is useful for delineating mega-environments using a formal statistical approach based on the factor analytic model. 

### Mixed Effect Linear-Bilinear Models

#### What if Genotypes or Environments, or both, are Random Effects? 

The basic linear mixed model used for fitting data from g genotypes, s sites, and r replicates when searching for subsets of environments and/or genotypes with non-COI is $Y=\mathrm{Xb}+Z_{r}r+Z_{g}g+e$ where **X **is the incidence matrix of 0s and 1s for the fixed effects of environments, and **Z**_r_ and **Z**_g_ are the incidence matrices of 0s and 1s for the random effects of replicates within environments and genotypes within environments, respectively. The random effect of genotypes within environments combines the main effects of genotypes and GE (GGE). Vector **b** denotes the fixed effects of environments and/or the effect of the design (i.e., replicates, incomplete blocks, etc.); vectors **r**, **g**, and **e** contain random effects of replicates within environments, genotypes within environments, and residuals within environments, respectively, and are assumed to be random and normally distributed with zero mean vectors and variance-covariance matrices **R**,** G,** and **E**, respectively. The variance-covariance matrices **R** and **E** are assumed to have the simple variance component structure, i.e., **R**= $\mathrm{diag}\left(\sigma_{r_{j}}^{2},j=1,2,...,s\right)⊗I_{r}$ and $\sigma_{e}^{2}⊗I_{\mathrm{rgs}}$ , where **I**_r_ and **I**_rgs_are identity matrices of order r and r×g×s, respectively, $\sigma_{r_{j}}^{2}$ , $\sigma_{e}^{2}$ are the replicates within the j^th^ environment and residual variances, respectively, and×is the Kronecker (or direct) product of the two matrices. The structure of **E **assumes that the residuals of the field plots at each environment (i.e., elements of vector **e**) are not spatially correlated; however, when field information is available, the spatial model approach using models such as the two-dimensional auto-regressive procedure in the direction of rows and columns in the field can be incorporated into the analyses. The solution ( $\overset{\wedge }{b}$ ) for the vector of fixed environment means and the vectors of random effects ( $\overset{\wedge }{r}$ and **ĝ**) are obtained from the mixed model equations.

The variance-covariance matrix **G** is indexed by two factors, environments and genotypes, and can therefore be written as the Kronecker product of two matrices indexing those factors, $G=\sum_{g}⊗I_{g}$ , where the j^th^ diagonal element of the s×s matrix $\sum_{g}$ is the genetic variance $\sigma_{g_{j}}^{2}$ within the j^th^ environment, and the jj’^th^ off-diagonal element is the genetic covariance $\rho_{\mathrm{jj}}\sigma_{g_{j}}\sigma_{g_{j'}}$ between environments j and j’; thus ρ_jj'_ is the correlation of genetic effects between environments j and j’. As for the genotype factor, the identity matrix **I**_g_ (of order g) is used when it is assumed that the genotypes are not related, and the breeding value of each genotype will be predicted only by the value of the empirical responses of the genotype itself. The environmental component of **G**, $\sum_{g}$ , can be modeled by the FA, whereas the genotypic component of **G** is modeled by the identity matrix **I**_g_, which assumes no relationship among genotypes. However, a relationship matrix **A** using the coefficient of parentage among genotypes can be used instead. 

#### The Factor Analytic model

The environmental component $\sum_{g}$ of the variance of random effects, **G**, can be modeled by the FA model, which expresses the random effect of the i^th^ genotype in the j^th^ environment as a linear function of latent variables x_ik_ with coefficients δ_jk_for k = (1, 2, … t), plus a residual η_ij_ that is $g_{\mathrm{ij}}=\sum_{k=1}^{t}x_{\mathrm{ik}}\delta_{\mathrm{jk}}+\eta_{\mathrm{ij}}$ where δ_jk_ is the factor loading of the j^th^ environment in the k^th^ latent factor, x_ik_ is the score of the i^th ^genotype in the k^th^ latent factor, and η_ij_ is the residual term. In matrix form the previous equation is expressed as $g_{\mathrm{ij}}=\left(\delta_{1}⊗I_{g}\right)x_{1}+\left(\delta_{2}⊗I_{g}\right)x_{2}+...+\left(\delta_{k}⊗I_{g}\right)x_{k}+\eta$ where vector $\left(\delta_{k}⊗I_{g}\right)x_{k}$ is of order gs × 1, and vector δ is of order gs × 1; then it can be written as $g=\left(\wedge ⊗I_{g}\right)x+\eta ,$ $\mathrm{where}\wedge =\left[\begin{array}{ccccc} \delta_{11} & \delta_{12} & . & . & \delta_{1k}\\ \delta_{21} & \delta_{22} & . & . & \delta k\\ . & . & . & . & .\\ . & . & . & . & .\\ \delta_{s1} & \delta_{s2} & . & . & \delta_{\mathrm{sk}} \end{array}\right]is a matrix of order s\times k$ with the k^th^ column containing the environment loadings for the k^th^ latent factor. Since it is assumed that the genotypes are unrelated, random effects **x** and **δ** are independent and have a joint normal distribution with a mean vector of zero and variances $V\left(x\right)=I_{K}⊗I_{g}=I_{\mathrm{kg}}$ $and V\left(\eta \right)=\Psi ⊗I_{g}$ (of order sg×sg), respectively, where **Ψ** is a diagonal matrix $\left(\sigma_{\eta_{1}}^{2},\sigma_{\eta_{1}}^{2},...,\sigma_{\eta_{s}}^{2}\right)$ of order s×s. Therefore, the variance of the random effects **g,** which separates the environmental and genotypic components, is given by

$\begin{array}{c} G=\left(\wedge ⊗I_{g}\right)V\left(x\right)\left(\wedge^{'}⊗I_{g}\right)+V\left(\delta \right)\\ =\left(\wedge ⊗I_{g}\right)\left(I_{k}⊗I_{g}\right)\left(\wedge^{'}⊗I_{g}\right)+\left(\Psi ⊗I_{g}\right)\\ \left({\wedge \wedge }^{'}+\Psi \right)⊗I_{g}=\mathrm{FA}(k)⊗I_{g} \end{array}$ where the elements of the matrixΛΛ^'^ $\wedge \wedge^{'}=\left[\begin{array}{ccccc} \delta_{11} & \delta_{12} & . & . & \delta_{1k}\\ \delta_{21} & \delta_{22} & . & . & \delta_{2k}\\ . & . & . & . & .\\ . & . & . & . & .\\ \delta_{s1} & \delta_{s2} & . & . & \delta_{\mathrm{sk}} \end{array}\right]\left[\begin{array}{ccccc} \delta_{11} & \delta_{21} & . & . & \delta_{\mathrm{s1}}\\ \delta_{12} & \delta_{22} & . & . & \delta_{s2}\\ . & . & . & . & .\\ . & . & . & . & .\\ \delta_{1k} & \delta_{2k} & . & . & \delta_{\mathrm{sk}} \end{array}\right]$ are estimates of the genetic variance within the j^th^ environment (i.e., diagonal elements of $\sum_{g},\sigma_{g_{j}}^{2}$ ) and estimates of the genetic covariances between the j^th^ and the j’^th^ environments (i.e., off-diagonal elements of $\sum_{g},\rho_{\mathrm{jj}}\sigma_{g_{j}}\sigma_{g_{j\text{'}}}$ ). 

Thus, FA can be interpreted as the linear regression of genotype and GE on latent environmental covariates (environmental loadings, δ_jk_), with each genotype having a separate slope (genotypic scores, x_ik_) but a common intercept (if main effects of genotypes are not distinguished from GE). The slopes of genotypes measure the sensitivity of the genotypes to hypothetical environmental factors represented by the loadings of each environment. 

A mixed-model analogue of AMMI or SREG has been developed using the factor analytic (FA) model for approximating the variance-covariance GE structure [12, 17, 14, 15]. Research conducted by Crossa *et al*. [16] and Burgueño *et al*. [19] described how to model variance-covariance GE and GGE using the FA model and how to incorporate the additive (relationship A) matrix and the additive × additive covariance matrix into the FA model based on pedigree information. Burgueño *et al*. [19] also described the equivalence between SREG2 and FA(2) for finding subsets of genotypes and environments without COI*.*

#### The Relationship Between the Factor Analytic and Sites Regression Models for Assessing Crossover genotype × Environment Interaction

In the FA model, the random effect of the i^th^ genotype in the j^th^ environment (g_ij_) is expressed as a linear function of latent variables x_ik_ with coefficients δ_jk_ for k=(1,2,…t), plus a residual, η_ij_, i.e., $\eta_{\mathrm{ij}},i.e,g_{\mathrm{ij}}=\mu_{j}+\sum_{k=1}^{t}x_{\mathrm{ik}}\delta_{\mathrm{jk}}+\eta_{\mathrm{ij}}$ so that the ij^th^ cell mean can be written as $y_{\mathrm{ij}}=g_{\mathrm{ij}}+\varepsilon_{\mathrm{ij}}$ . With only the first two latent factors being retained, g_ij_ is approximated by $g_{\mathrm{ij}}\approx \mu_{j}+x_{i1}\delta_{j1}+x_{i2}\delta_{j2}+\eta_{\mathrm{ij}}$ . Therefore, there is a clear connection between the SREG2 and FA(2) models. A similar connection between the AMMI2 and FA(2) models was established by Smith *et al*. [14].

Under principal component rotation, the directions and projections of the vectors of FA(2) and SREG2 in the biplot are the same. Therefore, the property of the SREG by which the first principal component of SREG2 accounts for non-crossover interaction (non-COI) and the second principal component of SREG2 is due to COI variability should hold for FA(2) as well. It should be pointed out that the absolute values of genotypic and environmental scores under the FA(2) and SREG2 models may not necessarily be the same; the estimates of the random effects in the FA(2) model are BLUPs (Best Linear Unbiased Predictions), whereas the estimates in the fixed effects SREG2 model are least squares estimates, that is, Best Linear Unbiased Estimates (BLUEs). Furthermore, the standard errors of the estimable functions of fixed effects under SREG differ from those of predictable functions of a mixture of fixed and random effects under FA, and FA models are more flexible in handling unbalanced data (the SREG model does not handle missing data). 

#### Prediction of Unobserved Individuals Based on Phenotypic Data While Modeling GE

Burgueño *et al*. [24] compared the predictive ability of linear mixed models when the GE is modeled by the FA model with that of simple linear mixed models when the GE is not modeled. A cross-validation scheme is used that randomly deletes some genotypes from environments for a 10-fold partition; the values for these genotypes are then predicted by the different models and correlated with their observed values in order to assess model accuracy. A total of six multi-environment trials (one potato trial, three maize trials, and two wheat trials) with GE of varying complexity were used in the evaluation. Although Burgueño *et al*. [24] analyzed six MET, here we present results from only three maize METs (M1-MET, M2-MET, and M3-MET). The models used for prediction are linear mixed models 1 and 2, which are two simple mixed models that had the random GE term not modeled, and two linear models, 3FA and 4FA, which had the GE modeled by the FA model (Table **1**).

For M1-MET, the best predictive model was model 3FA, which had a correlation of 0.878 that represents a 6% increase in accuracy with respect to simple linear mixed model 1, whereas model 4FA, with a correlation of 0.867, had a 4.7% increase in accuracy with respect to the simple linear mixed model 1 (Table **2**). Prediction of the overall genotypic effects of the simple linear mixed models in a complex GE setting was poor as compared with that of models that considered covariances between environments. The correlations between the observed and predicted values of the four models fitted to M2-MET and M3-MET were higher in the M2-MET than in the M3-MET because the GE in M2-MET was simpler than the GE in M3-MET. For M2-MET, the two FA models (3FA and FA) did show slightly better predictability (0.938) than their non-FA counterparts (models 1 and 2) (0.916) (Table **2**), and there was a 2.4% increase in the predictive ability of these models with respect to simple linear model 1. For M3-MET, the best predictive models were 3FA and 4FA, with correlations of 0.848 and 0.852, respectively. However, the percentage increases in correlations of these models over model 1 were 2.9% and 3.4% for models 3FA and 4FA, respectively, indicating that for more complex GE (M3-MET), the FA models increase the predictability more than the simple linear mixed model for less complex GE (M2-MET) (Table **2**).

#### Incorporating Pedigree Information into Linear-Bilinear Mixed Models While Modeling GE 

As has been shown, the linear mixed version of SREG and AMMI naturally leads to a FA form for the genetic variance-covariance for environments that is more parsimonious and flexible than other variance-covariance structures. Since the above mentioned models are linear mixed models, they also have the usual advantages when compared with ordinary fixed effects linear-bilinear AMMI and SREG models. That is, error variance modeling can be accommodated, in particular, heterogeneity of block and error variance between environments and within-environment spatial correlation, and incomplete data are handled with ease. Furthermore, when genotypes are considered as random effects, the coefficients of parentage can be incorporated into the FA for modeling GE or GGE of the mixed versions of AMMI and SREG, respectively, hence obtaining more precise estimates of the breeding values of genotypes. The genetic covariance between any pair of related individuals (i and i’), due to their additive genetic effects, is equal to two times the coefficient of parentage (COP=**f**_ii’_), also known as the coefficient of coancestry, times the additive genetic variance, i.e., 2**f**_ii*’*_ $\sigma_{a}^{2}$ =**A** $\sigma_{a}^{2}$ , where **A** is the additive relationship matrix. In self-pollinated species, **A** $\sigma_{a}^{2}$ is the variance-covariance matrix of the breeding values (additive genetic effects). Closely related individuals contribute more to the prediction of breeding values of their relatives than do less closely related genotypes. Moreover, when one genotype is missing (either partially or totally), its breeding value can still be predicted from its relatives, albeit less efficiently than if the data were complete.

A linear mixed model used for fitting the data from g genotypes, s sites and r replicates, assuming the relationship of the genotypes is measured by the matrix COP=**f**_ii’_ (of order g), is 

$\left[\begin{array}{c} y_{1}\\ y_{2}\\ .\\ .\\ .\\ y_{s} \end{array}\right]=\left[\begin{array}{c} {1\mu }_{1}\\ {1\mu }_{2}\\ .\\ .\\ .\\ {1\mu }_{s} \end{array}\right]+\left[\begin{array}{cccccc} Z_{R_{1}} & 0 & . & . & . & 0\\ 0 & Z_{R_{2}} & . & . & . & 0\\ . & . & . & . & . & .\\ . & . & . & . & . & .\\ . & . & . & . & . & .\\ 0 & . & . & . & . & Z_{R_{S}} \end{array}\right]r+\left[\begin{array}{cccccc} Z_{G_{1}} & 0 & . & . & . & 0\\ 0 & Z_{G_{2}} & . & . & . & 0\\ . & . & . & . & . & .\\ . & . & . & . & . & .\\ . & . & . & . & . & .\\ 0 & . & . & . & . & Z_{G_{S}} \end{array}\right]g+e.$

where y_j_ is the vector of the response variable in the j^th^ site (j=1,2,…,s), **1** is a vector of ones, µ_j_ is the population mean of the j^th^ site, and $Z_{R_{j}}$ and $Z_{G_{j}}$ are the design matrices of the random effects of replicates and genotypes within the j^th^ site, respectively. The variance-covariance matrices **R** and **E** are assumed to have the simple variance component structure $R=\sum_{r}⊗I_{r}$ and $E=\sum_{e}⊗I_{\mathrm{rg}}$ , where **I**_r_ and **I**_rg_ are the identity matrices of orders r and r×g, respectively; the **∑**_r_= $\mathrm{diag}\left(\sigma_{r_{j}}^{2},j=1,2,...,s\right)$ and **∑**_e_= $\mathrm{diag}\left(\sigma_{e_{j}}^{2},j=1,2,...,s\right)$ are the s×s replicate and error variance-covariance matrices among pairs of s sites, respectively; $\sigma_{r_{j}}^{2}$ , $\sigma_{e_{j}}^{2}$ are the replicate and residual variances within the j^th^ site, respectively; and ⊗ is the Kronecker (or direct) product of the two matrices. In covariance pattern models, it is assumed that residuals have a multivariate normal distribution with zero means and covariance matrix **E**. In this study, the structure of **E **assumes that the residuals of the field plots at each site (i.e., elements of vector e) are not spatially correlated, that is, $E=\sum_{e}⊗I_{\mathrm{rg}}$ . However, when the field location of the plots is recorded, the matrix **E** could be modeled as a structure that is less restrictive than $\sum_{e}⊗I_{\mathrm{rg}}$ . Commonly, these spatial correlations among field plots are modeled using the two-dimensional auto-regressive procedure in the direction of the rows and columns in the field. The design effects given by the replicates and the incomplete blocks within replicates in environments can be easily incorporated into the above linear mixed model as random or fixed effects. 

Vectors **r**, **g**, and **e** contain random effects of replicates within sites, genotypes within sites, and residuals within sites, respectively, and are assumed to be random and normally distributed with zero mean vectors and variance-covariance matrices R, G, E, respectively, such that $\left[\begin{array}{c} r\\ g\\ e \end{array}\right]∼N\left(\left[\begin{array}{c} 0\\ 0\\ 0 \end{array}\right]'\left[\begin{array}{ccc} R & 0 & 0\\ 0 & G & 0\\ 0 & 0 & E \end{array}\right]\right)$ .

The variance-covariance matrix **G**, which combines the main effect of genotypes (breeding values) and GE, can be represented as $G=\sum_{g}⊗A$ , where the j^th^ diagonal element of the s×s matrix ∑_g_ is the additive genetic variance $\sigma_{a_{j}}^{2}$ within the j^th^ site, and the jj’^th^ element is the additive genetic covariance ρjj'σajσaj' between sites j and j’; thus ρ_jj'_ is the correlation of additive genetic effects between sites j and j’. The variance-covariance matrix **G** can be modeled using the FA structure. The matrix **A**=2f_ii’_ is of order g×g and measures the relationship or covariance between relatives due to additive genetic effects. When genotypes are not related, **A** is replaced by **I**_g_ (identity matrix of order g) [14, 45] and the breeding value of each genotype will be predicted only by the value of the empirical response of the genotype itself.

An example of how to incorporate pedigree information while modeling GE using the FA is described by Crossa *et al*. [16] using data from a CIMMYT bread wheat (*Triticum aestivum *L.) international trial. Twenty-nine lines (1-29) were tested in 16 international sites, namely Mexico (MEX), USA (two sites, USA1 and USA2), Turkey (TKY), Israel (ISR), Bangladesh (BGD), India (IND), Pakistan (PKT), Syria (SYR), Spain, (SPN) (two sites, SPN1 and SPN2), Nepal (NPL), Kenya (KNY), Zimbabwe (ZBW), New Zealand (NZL), and Chile (CHL), in randomized complete block designs with three replications at each site. The response variable analyzed was grain yield (Mg ha^-1^). There were five sets of sister lines (5, 6), (4, 19, 20), (14, 15), (21, 23, 24), and (28, 29).

**Fig. (1):**
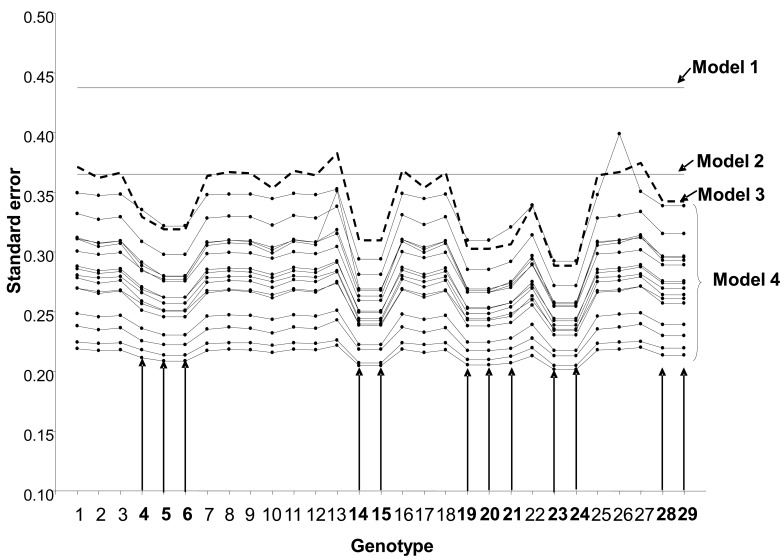
Standard errors of BLUPs of 29 wheat genotypes (1-29) within 16 sites for grain yield for four linear-bilinear models (models 1, 2, 3, and 4). Sister lines are in bold (5,6), (4,19,20), (14,15), (21,23,24), (28,29). For model 15, each graph line represents one of the 16 sites. Model 1 is the standard fixed linear-bilinear model (SREG), model 2 is a simple mixed linear-bilinear model without **A** (not modeling GE), model 3 is a simple mixed linear-bilinear model using **A** (not modeling GE), and model 4 is a mixed linear-bilinear model that models the GE using the FA and includes information on relatives (**A**) (adapted from Crossa *et al.* [16]).

Standard errors (SE) of BLUPs of breeding values of the lines for grain yield were smaller for models using information on relatives and when the main effect of genotypes and GE were modeled using FA structure of the **G** variance-covariance matrix (Fig. **1**). The standard fixed linear-bilinear model 1 where all the effects are considered as fixed effects had the largest SEs, followed by a simple mixed linear-bilinear model 2 (with the GE considered as random effect) without **A **(without modeling GE) and a simple mixed linear-bilinear model 3 with **A** (without modeling GE). In contrast, the mixed linear-bilinear model 4 that models the GE using the FA and includes information on relatives (**A**) had the smallest SE for most of the line-environment combinations. The lines in Fig. (**1**) (from top to bottom) graph the SEs of BLUPs of breeding values of the 29 genotypes computed from models 1, 2, 3, and 4 in each of the 16 sites included in the trial (NZL, USA1, TKY, ZBW, CHL, SPA2, MEX, SYR, ISR, SPA1, NPL, KNY, IND, PKT, BGD, and USA2). New Zealand (NZL) had the largest SE of the BLUPs of the breeding values and USA2 had the smallest. Only the SE of the BLUP of genotype 26 in NZL from model 4 was larger than those from models 2 and 3 (Fig. **1**). The benefits, in terms of precision, of including information on related lines are evident when comparing models 2 and 3; model 3 is similar to model 2, but includes information on relatives. It is clear that the SEs of sister lines were always smaller than the SEs of genotypes that had no relatives in the trial in all cases except for models 1 and 2, which do not incorporate this information. When modeling the main effects of genotype and GE in conjunction with the information on relatives, the improvement in the precision of the BLUP of the sisters, as well as of the other lines, is evident. It should be pointed out that no model is selected based on the SE of the means because SEs are model-dependent and one might be dealing with an inappropriate model despite its small SE. Fig. (**1**) only shows the natural impact of performing a more realistic statistical analysis instead of ignoring the relationship between genotypes and environments.

**Fig. (2):**
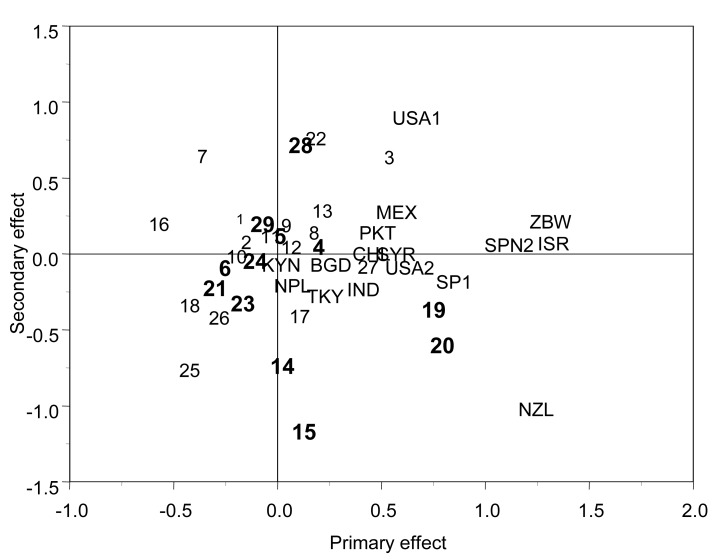
Biplot of the standard fixed effect linear-bilinear model 1 for grain yield. Lines are 1-29. Sister lines are in bold (5,6), (4,19,20), (14,15), (21,23,24), (28,29). The 16 sites are MEX, USA1, USA2, TKY, ISR, BGD, IND, PKT, SYR, SPN1, SPN2, NPL, KNY, ZBW, NZL, and CHL (adapted from Crossa *et al.* [16]).

The standard biplots of a fixed effect linear-bilinear model (model 1 that excludes matrix **A**) and model 4 (FA including matrix **A**) are depicted in Figs. (**2** and **3**), respectively. The biplot of model 1 (Fig. **2**) is the usual biplot of a fixed SREG model that shows genotypes 19 and 20 as having a positive response in terms of genotype main effect and GE for most of the sites because they are in the same direction. Genotypes 16, 18, and 25, located on the opposite side of the biplot, have a negative response in all sites. Sites located farther away from the center, such as NZL, USA1, ZBW, ISRL, and SPN2, are the ones that discriminate the genotypes the most. Pairs of sister lines 19 and 20, and 14 and 15 are distinct from the others. Sister lines are scattered throughout the two dimensions of the biplot. 

**Fig. (3):**
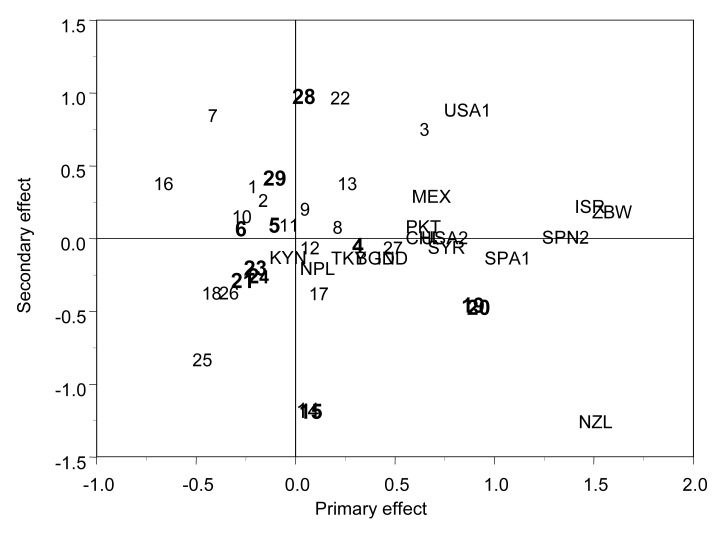
Biplot of the mixed linear-bilinear model 4 with FA for GE and pedigree information (A) for grain yield. Lines are 1-29. Sister lines are in bold (5,6), (4,19,20), (14,15), (21,23,24), (28,29). The 16 sites are MEX, USA1, USA2, TKY, ISR, BGD, IND, PKT, SYR, SPN1, SPN2, NPL, KNY, ZBW, NZL, and CHL (adapted from Crossa *et al.* [16].

When the main effect of genotypes and GE are modeled together using the factor analytic variance-covariance model 4 (FA that includes information between relatives (Fig. **3**)), sister lines with a strong genetic association are held together, but others, such as genotype 4, tended to be farther away from their sister lines (19 and 20), because their COP was 0.792; a similar situation occurred with sister lines 28 and 29, with a COP of 0.774. Although the general patterns of response in terms of directions and projections of genotypes and sites in the biplot were not altered by using different models, the inclusion of information between relatives in conjunction with modeling the main effects of genotypes and GE by means of a factor analytic structure offers the best option for predicting breeding values of genotypes across different environments and studying the main effects of genotypes and GE. Models with FA for GE and pedigree information gave a more realistic view of the relationship between the genotypes themselves, between sites, and between their interactions. 

## STATISTICAL MODELS FOR MAPPING QTLS AND STUDYING QTL × ENVIRONMENT INTERACTION (QEI) 

This section is related to the issue of understanding the nature and causes of interaction. The factorial regression (FR) model (e.g., Vargas *et al*. [46] and van Eeuwijk *et al*. [47]) is useful for studying the effects of both genetic and environmental covariables and for developing functional relationships and predictability with explanatory covariables. In plant breeding, much research is directed at locating regions of the chromosomes that are involved in the physiological processes underlying phenotypical traits. These regions are called quantitative trait loci (QTLs). When the relative contributions of these regions to the explanation of the phenotype differ between genotypes, then QTL × environment interaction (QEI) occurs. The statistical problem can be interpreted as a multivariate multiple regression of phenotypic traits as observed over a set of environments on a set of genetic predictors. FR provides a suitable framework for QEI analysis. Crossa *et al*. [48] give examples of how FR can be used for assessing the chromosomal location of QTLs and QEI and the importance of their effects.

There are approaches in which the GE is modelled directly using regression on environmental (and/or genotypic) variables, rather than regression on the environmental mean, as originally proposed by Yates and Cochran [37]. A useful linear model for incorporating external environmental (or genotypic) variables is the FR model [49, 50]. The FR models are ordinary linear models that approximate the GE effects of Eq. 1 by the products of one or more of the following: (1) genotypic covariables (observed) × environmental potentialities (estimated); (2) genotypic sensitivities (estimated) × environmental covariables (observed); (3) scale factor (estimated) × genotypic covariables (observed) × environmental covariables (observed). The aim of FR is to replace, in the GE subspace, genotypic and environmental factors with a small number of genotypic and environmental covariables. Vargas *et al*. [51] further developed the statistical approaches described by Crossa *et al*. [48] and van Eeuwijk *et al*. [52] for modeling QTLs and QEI. The main objectives of their research were to demonstrate the use of: (1) FR for estimating effects and locations of QTL and QEI, and (2) FR for modeling and interpreting QEI in terms of products of genetic predictors and environmental variables.

In FR, genotypic covariables x_a_ (a =1…A), with values x_ia_ for the i^th^ genotype can be introduced for the genotypic main effect, $G_{i}=x_{\mathrm{ia}}\rho_{a}+\mathrm{residual}$ where ρ_a_ is the regression coefficient for the regression of G_i_ on the genotypic covariables, x_ia_. For more than one genotypic covariable, this becomes $G_{i}=\sum_{a=1}^{A}x_{\mathrm{ia}}\rho_{a}+\mathrm{residual}$ . In the context of molecular markers and when attempting to map QTLs, the genotypic covariables, x_a_, are replaced by the genetic predictors x_q_, and the FR framework can also be used to do a genome scan for QTL effects. Analogous to the genotypic main effect, in FR, the environmental main effect, E_j_, can also be regressed on environmental covariables z_b_ with values z_jb_ for the j^th^ environment. The corresponding partitioning is $E_{j}=z_{\mathrm{jb}}\beta_{b}+\mathrm{residual}$ for one environmental covariable, or $E_{j}=\sum_{b=1}^{B}z_{\mathrm{jb}}\beta_{b}+\mathrm{residual}$ for multiple environmental covariables. The parameter β_b_ is the regression coefficient for the regression of the environmental main effect on the environmental covariables, z_jb _for the jth environment.

Within a QTL analysis by FR, a multiple QEI model follows easily from models for GE: ${\left(\mathrm{GE}\right)}_{\mathrm{ji}}=\sum_{q=1}^{Q}x_{\mathrm{iq}}\rho_{\mathrm{jq}}+\mathrm{residual}$ where ρ_jq_ represents a QEI effect, i.e., a differential QTL expression in relation to the main effect QTL expression, for the q^th^ QTL in j^th^ environment. QEI for a QTL q’ can be further modeled by regressing it on an environmental covariable, z_b_: ${\left(\mathrm{GE}\right)}_{\mathrm{ij}}={\nu_{q'b}x}_{\mathrm{iq}}\rho_{\mathrm{jb}}+\mathrm{residual}$ . For multiple QTLs, this generalizes to ${\left(\mathrm{GE}\right)}_{\mathrm{ij}}=\sum_{q=1}^{Q}\sum_{b=1}^{B}{\nu_{\mathrm{qb}}x}_{\mathrm{iq}}\rho_{\mathrm{jb}}+\mathrm{residual}$ . One or more QTL main effects can be tested by comparing the model ${\bar{y}}_{\mathrm{ij}}=\mu +\sum_{q=1}^{Q}x_{\mathrm{iq}}\rho_{q}+E_{j}+\mathrm{residual}$ with the model ${\bar{y}}_{\mathrm{ij}}=\mu +E_{j}$ . When main effect QTL expression and QEI are considered together, this is equivalent to fitting different effects for the same QTL in different environments. A specific test for QE compares ${\bar{y}}_{\mathrm{ij}}=\mu +\sum_{q=1}^{Q}x_{\mathrm{iq}}\rho_{q}+E_{j}+\sum_{q=1}^{Q}x_{\mathrm{iq}}\rho_{\mathrm{jq}}+\mathrm{residual}$ to ${\bar{y}}_{\mathrm{ij}}=\mu +\sum_{q=1}^{Q}x_{\mathrm{iq}}\rho_{q}+E_{j}+\mathrm{residual}$ . F-tests can be constructed from ratios of regression mean squares to independent error term.

An example including 211 F2-derived F3 maize families from a biparental cross evaluated across eight environments differing in the level of drought stress and soil nitrogen content is employed to illustrate the use of the fixed effect FR linear model for mapping QTLs and for studying the influence of external covariables on QEI [51]. Table **3** shows parts of the analysis of variance table at position 63 cM on chromosome 10 [51]. The first part shows the usual analysis of variance for a two-way table of grain yield measured in 211 genotypes with partitioning of the joint effect of G+GE into G and GE effects. The middle part of Table **3** shows the variability due to QTL+QE effects in parts of the genome other than chromosome 10 (i.e., due to QTLs on chromosomes 1-9), the variability due to G+GE after correction for QTLs on the other chromosomes, and the corresponding partitioning into G and GE components. The last part shows the partitioning of G+GE adjusted for the QTLs on chromosomes 1-9 into variation due to QTL+QE at position 63 cM on chromosome 10 and deviations from the QTL model. When the QEI effects were regressed on the set of environmental covariables, maximum temperature at flowering showed the closest relationship with the QEI effects and explained 23.8% of the QEI. The effect of this environmental covariable was highly significant by an F-test for the regression mean square over the deviations from the regression. 

For grain yield, Fig. (**4**) depicts the profile of R^2^_QTL_, R^2^_QEI_, and R^2^_QTL+QEI_ and the corresponding critical values for α=0.01 based on 1000 randomizations. There is good reason to believe that there are environment-specific QTLs around 63 cM of chromosome 10 (QTL+QEI and QEI effects were both significant). In contrast, only main effect QTLs were observed in other chromosomes. 

### Linear Mixed Models for Multi-Trait Multi-Environment QTL Analysis

Plant breeders are interested in evaluating genotypes for multiple traits. A general formulation of a linear mixed model for the multi-trait multi-environment (MTME) model is presented by Malosetti *et al*. [53]. The initial model is y=Xβ+Zu+e where the response variable **y** is modelled by fixed and random effects factors** β **and **u**, respectively; **X** and **Z** are design matrices assigning fixed and random effects to the observations, respectively. Random genetic effects are assumed to be normally distributed, **u**~N(**0**,**G**), with **G **the genetic (co)variance matrix. Finally, **e** is a vector of non-genetic residuals associated with each observation and normally distributed, **e**~N(**0**, **R**). The phenotypic (co)variance is given by V(**y**) = **ZGZ**’ + **R**. From a breeder’s point of view, **G** is of special interest, as it reflects the magnitude and pattern of relationships between genetic effects of traits. Exploiting the genetic correlations between traits is useful because it may be a primary trait with low heritability or a difficult-to-measure primary trait that is correlated with an easy-to-measure secondary trait with high heritability.

A QTL model arises by including the effect of a putative QTL as follows: $y=X\beta +X^{\mathrm{QTL}}\alpha +Zu^{*}+e$ . The extra term in the model is composed of a design matrix **X^QTL^,** which is derived from molecular marker information and a vector of fixed QTL effects (**α**). In an MTME model, vector **α** has dimensions JK×1 and contains the additive genetic QTL effects for all the traits in each of the environments. The random genetic effects, now collected in a vector u*, result from the effects of QTLs outside the test region, that is, the genetic background. Genetic background effects are assumed to be normally distributed: **u **~ N(0, G*). Note that G* represents the part of the genetic (co)variance that is not explained by the QTL. The extension from a single-QTL model to a multi-QTL model is straightforward and given by

$y=X\beta +\sum_{q=1}^{Q}X_{q}^{\mathrm{QTL}}\alpha^{q}+Zu^{*}+e$ . 

**Fig. (5):**
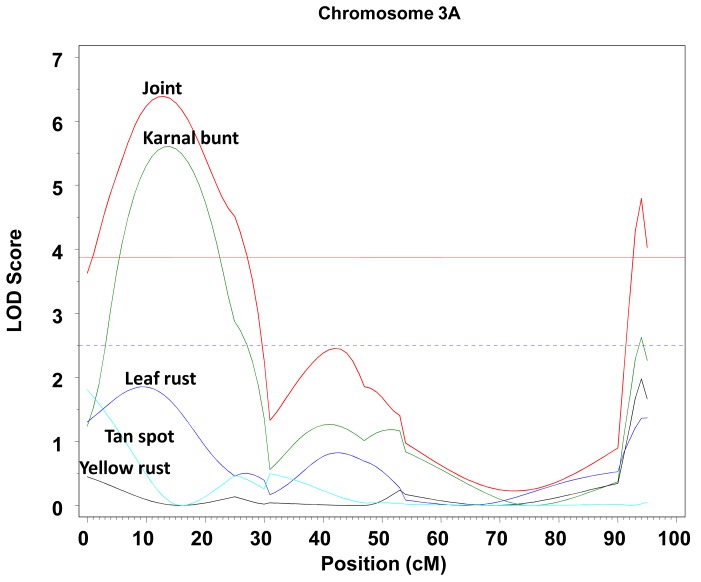
Profile of LOD of chromosome 3A from the multi-trait analysis. The LOD profile of the joint analysis of all four traits (red) and the LOD profile of the marginal analyses for Karnal bunt (green), leaf rust (blue), tan spot (light blue), and yellow rust (black). The LOD threshold for the joint profile is the red horizontal line and the LOD threshold for the traits is the blue horizontal line.

**Fig. (6):**
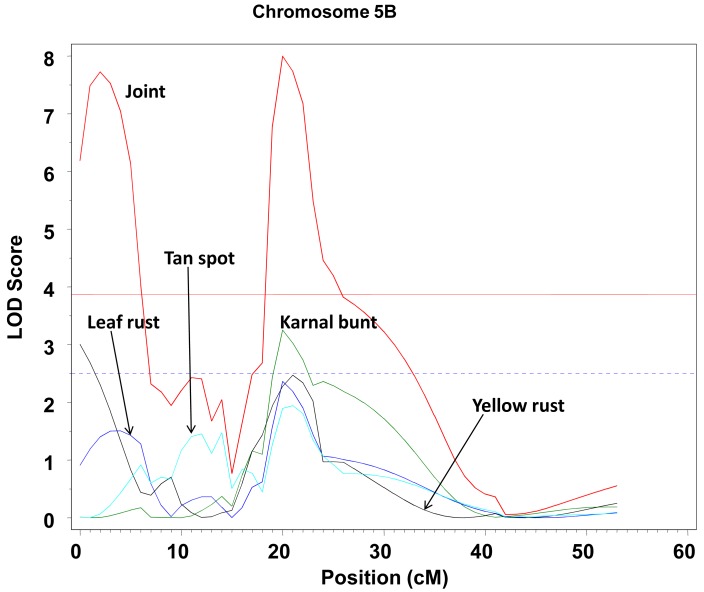
Profile of LOD of chromosome 5B from the multi-trait analysis. The LOD profile of the joint analysis of all four traits (red) and the LOD of the marginal analyses for Karnal bunt (green), leaf rust (blue), tan spot (light blue) and yellow rust (black). The LOD threshold for the joint profile is the red horizontal line and the LOD threshold for the traits is the blue horizontal line.

An example of the application of the MTME is presented in a wheat trial (S. Singh and M. Vargas, personal communication), where four diseases (Karnal bunt, tan spot, yellow rust, and leaf rust) were simultaneously measured in a biparental progeny. A multi-trait QTL scan was performed and the various significant QTLs detected are depicted in Figs. (**5** and **6**). Fig. **5** shows the joint profile and the two significant peaks for the same trait, Karnal bunt, one between 20 and 20 cM and the other one at about 95 cM chromosome 3A. On the other hand, Fig. **6** shows two significant peaks for two different traits, one for yellow rust at the beginning of chromosome 5B and another peak for Karnal bunt at about 20 cM. These findings suggest that some diseases are determined in similar regions of the chromosomes; they should be studied jointly rather than by single-trait QTL mapping.

## STATISTICAL MODELS FOR GENOMIC SELECTION AND PREDICTION 

Selection in plant breeding is based on estimates of breeding values obtained with pedigree-based mixed models [16-20]. These models have been used successfully for predicting breeding values in plants and animals. However, pedigree-based models cannot account for Mendelian segregation, a term that under an infinitesimal additive model [23] and in the absence of inbreeding, explains one half of the genetic variability. Molecular markers (MM) allow tracing Mendelian segregation at several positions of the genome; potentially, this may increase the accuracy of estimates of genetic values and of the genetic progress attainable when these predictions are used for selection purposes.

Genomic selection (GS) (or genome-wide selection) is an approach for improving quantitative traits [29, 35] that uses all available MM across the genome to estimate genetic values. Genomic selection has been validated in several species and populations in animal breeding [35, 36]. However, reports on the use of GS in plants are few and refer mainly to computer simulation studies such as the research of Bernardo and Yu [30], who concluded that GS was superior to marker assisted selection in maize. In recent articles, de los Campos *et al*. [31], Pérez *et al*. [34], and Crossa *et al*. [32, 33] validated GS in plant breeding using genomic regression and showed that models using MM were more accurate in predicting grain yield in wheat and maize than those based on pedigree only. 

One method for incorporating markers into models for GS is to define genetic values (g_i_ , i=1,...,g) as a parametric regression on marker covariates x_ij_ (which can take values of 1, 0 or -1 for a biallelic marker of a segregating population, or values of 1 and 0 for inbred lines) of the form $g_{i}=\sum_{j=1}^{p}x_{\mathrm{ij}}\beta_{j}$ . Phenotypes (y_i_) can then be represented as $y_{i}=\sum_{j=1}^{p}x_{\mathrm{ij}}\beta_{j}+\varepsilon_{i}$ (j=1,2,…,p), whereβ_j_ is the regression of y_i_ on the j^th^ marker covariate, and ε_i_ is a model residual. This approach was first proposed by Meuwissen *et al.* [29] and is the most commonly used in GS. With high-density markers, the number of markers exceeds the number of individuals, and estimation of marker effects via ordinary least squares (OLS) is not feasible. Instead, penalized or Bayesian estimation methods are commonly used. Several penalized and Bayesian shrinkage estimation methods are available. Examples of the first group are Ridge Regression (RR) and the Least Absolute Shrinkage and Selection Operator (LASSO) of Tibshirani [54] and Elastic Net (EN). The list of Bayesian models is extensive and includes Bayesian counterparts of RR (BRR) and LASSO [55] (BL) and the Elasctic Net (BEN). 

We considered models for GS that differ in the information used (pedigree, molecular markers or both) and in the way molecular markers are incorporated into the model (parametric regression or semi-parametric regression). In all cases, the response variable is the average standardized performance of each line within each environment, that is ${\bar{y}}_{i}=\sum_{k=1}^{n_{i}}y_{\mathrm{ik}}/\left(\mathrm{SD}\times n_{i}\right)$ , where n_i_ is the number of replicates available for the i^th^ line and SD is the (sample) standard deviation of the response variable $\left\{{\bar{y}}_{i}\right\}$ . The conditional distribution of this phenotype given µ, **g**, $\sigma_{\varepsilon }^{2}$ is Gaussian, $p\left({\left.\bar{y}\right|\mu ,g,\sigma }_{\varepsilon }^{2}\right)=\prod_{i=1}^{n}N\left(\left.{\bar{y}}_{i}\right|\mu +g_{i},\sigma_{\varepsilon }^{2}/n_{i}\right)$ where µ is an intercept, $\bar{y}=\left\{{\bar{y}}_{i}\right\}$ and $g=\left\{g_{i}\right\}$ are vectors of average phenotypes and genetic values, respectively, and $\sigma_{\varepsilon }^{2}$ is a residual variance. 

A standard additive infinitesimal model (e.g., Fisher [25] and Henderson [57]) postulates that genetic values are multivariate normal, centered at zero, and with a co-variance matrix proportional to the numerator relationship matrix (**A**) computed from the pedigree, that is, g_i_ = a_i_, where $a=\left(a_{1},...,a_{n}\right)∼N\left(\left.a\right|0,A\sigma_{a}^{2}\right)$ , and $\sigma_{a}^{2}$ is the additive variance. The collection of unknowns in this model is $\left\{{{\mu ,a,\sigma }_{a}^{2},\sigma }_{\varepsilon }^{2}\right\}$ and in a Bayesian setting, a prior density is assigned to these unknowns. Following standard assumptions, independent scaled inverse Chi-square distributions for the residual and the additive variance are chosen. The joint prior of this pedigree-based model becomes: $p\left(\mu ,a,\sigma_{\varepsilon }^{2}\left.\sigma_{a}^{2}\right|{\mathrm{df}}_{\varepsilon },S_{\varepsilon },{\mathrm{df}}_{A},S_{a}\right)\propto N\left(\left.a\right|0,A\sigma_{a}^{2}\right)$ $x^{-2}\left(\left.\sigma_{\varepsilon }^{2}\right|{\mathrm{df}}_{\varepsilon },S_{\varepsilon }\right)x^{-2}\left(\left.\sigma_{a}^{2}\right|{\mathrm{df}}_{a},S_{a}\right)$ where $x^{-2}\left(\left..\right|.,.\right)$ is a scaled-inverse Chi-square density, and df and S are prior degree-of-freedom and scale parameters, respectively. The joint posterior distribution for this model is obtained combining the likelihood and prior, such that: $\begin{array}{c} p\left(\mu ,a,\sigma_{\varepsilon }^{2},\left.\sigma_{a}^{2}\right|\bar{y},{\mathrm{df}}_{\varepsilon },S_{\varepsilon },{\mathrm{df}}_{a},S_{a}\right)\propto \prod_{i=1}^{n}N\left(\left.{\bar{y}}_{i}\right|\mu +a_{i},\sigma_{\varepsilon }^{2}/n_{i}\right)\\ N\left(\left.a\right|0,A\sigma_{a}^{2}\right)x^{-2}\left(\left.\sigma_{\varepsilon }^{2}\right|{\mathrm{df}}_{\varepsilon },S_{\varepsilon }\right)x^{-2}\left(\left.\sigma_{a}^{2}\right|{\mathrm{df}}_{a},S_{a}\right) \end{array}$

Inferences are based on samples obtained using a Gibbs sampler. 

An alternative is to replace **A** with kinship matrix (**U**) estimated using marker genotypes. A kinship-based infinitesimal model (K) is obtained using g_i_ = u_i_ , where $u={\left(u_{1},...,u_{n}\right)}^{'}∼N\left(\left.u\right|0,U\sigma_{u}^{2}\right)$ and $\sigma_{u}^{2}$ is the associated variance parameter.

### Penalized Parametric Regression on Marker Effects

In ridge regression, estimates are obtained by minimizing the residual sum of squares, $\min_{\beta }\left\{\sum {\left(y_{i}-x_{i}^{'}\beta \right)}^{2}\right\}$ subject to the following constraint: $\sum_{j}\beta_{j}^{2}\leq t$ or, equivalently, ${\overset{\wedge }{\beta }}_{\mathrm{RR}}=\arg_{\beta }\min\left\{\sum_{i=1}^{n}{\left(y_{i}-x_{i}^{'}\beta \right)}^{2}+\lambda \left(t\right)\sum_{j}\beta_{j}^{2}\right\}$ .

The solution to this optimization problem can be shown to be $\overset{\wedge }{\beta }={\left(X^{'}X+\lambda \left(t\right)I\right)}^{-1}X^{'}y$ , where λ(t) > 0 is a regularization parameter that induces shrinkage of estimates of effects towards zero and controls the trade-off between goodness of fit amd model complexity. Even though these estimates are biased, the sampling variance is reduced, yielding smaller mean-squared error and better predictive ability.

An alternative to ridge regression is to use LASSO [54]. Estimates in LASSO are obtained by minimizing the residual sum of squares, $\min_{\beta }\left\{\sum {\left(y_{i}-x_{i}^{'}\beta \right)}^{2}\right\}$ subject to the following constraint: $\sum_{j}\left|\beta_{j}\right|\leq t$ or, equivalently, ${\overset{\wedge }{\beta }}_{\mathrm{LASSO}}=\underset{\beta }{\arg\min}\left\{\sum_{i=1}^{n}{\left(y_{i}-x_{i}^{'}\beta \right)}^{2}+\lambda \left(t\right)\sum_{j}\left|\beta_{j}\right|\right\}.$ . 

Unlike the quadratic penalty of ridge regression (L_2_ norm, $\sum_{j}\beta_{j}^{2}$ ) the absolute-value penalty of LASSO (L_1_ norm, $\sum_{j}\left|\beta_{j}\right|$ ) induces selection and shrinkage simultaneously as some predictor effects may take the value of zero. When predictors are highly co-linear empirical evidences show that RR has better prediction accuracy than LASSO; this situation is common in GS with dense molecular markers.

One variant of the traditional LASSO is the elastic net LASSO, which differs from LASSO in that it uses two penalties the L_2_ and the L_1_ norms such that ${\overset{\wedge }{\beta }}_{\mathrm{EN}}=\underset{\beta }{\arg\min}\left\{\sum_{i=1}^{n}{\left(y_{i}-x_{i}^{'}\beta \right)}^{2}+\lambda_{1}\left(t\right)\sum_{j}\left|\beta_{j}\right|+\lambda_{2}\left(t\right)\sum_{j}\beta_{j}^{2}\right\}$

The advantage of the EN is that, by adding another penalty, it stabilizes the LASSO solution when some predictors are highly correlated; such is the case of MMs used in GS. Two other variants of LASSO are the group LASSO and the fused LASSO. Group LASSO selects variables at a group level such that some groups of predictors are selected together; this may be useful when the researcher, instead of examining the effect of individual molecular markers wishes to examine the effects of haplotypes (genes) comprising several molecular markers in high linkage disequilibrium. Group LASSO could also be useful when there are more than two alleles for each molecular marker and the breeder wishes to keep all the alleles of the same MM active in the model. The fused LASSO focuses on adjacent predictors such that the value effects tend to be the same for adjacent predictors. This can be useful when there is a natural ordering of predictors, for example, when markers have been ordered on a common map. 

### Bayesian Shrinkage Regression Methods

Estimates of regression coefficients derived from penalized optimization problems such RR, LASSO or EN are equivalent to posterior modes in certain class of Bayesian Models. In the Bayesian approach, inferences are based on the posterior distribution of the unknowns (hyper-parameters, H) given the data (**y**), p(H|**y**). Following Bayes’ rule, this density is proportional to the product of the conditional distribution of the data given the unknowns, p(**y**|H), or the Bayesian likelihood times the prior density assigned to model unknowns p(H). In the models we are interest, the conditional distribution of the data given the parameters p(**y**|H) can be represented as the the product of independent normal densities centered at the regression function, $E\left(y_{i}|x_{i},\beta \right)=\sum_{j=1}^{p}x_{\mathrm{ij}}\beta_{j}$ and with common residual variance $\sigma_{\varepsilon }^{2}$ , that is $p\left(y|x,\beta ,\sigma_{\varepsilon }^{2}\right)=\prod_{i}N\left(y_{i}|\sum_{j}x_{\mathrm{ij}}\beta_{j},\sigma_{\varepsilon }^{2}\right)$

Marker effects are assigned identical and independent normal prior densities, $p\left(\beta |\omega \right)=\prod_{j}N\left(\beta_{j}|\omega \right)$ whereωrepresents hyper-parameters indexing the prior density of marker effects. Following Bayes’ rule, the posterior distribution is:

$\begin{array}{c} p\left(\beta |y,\sigma_{\varepsilon }^{2},\omega \right)\propto p\left(y|X,\beta ,\sigma_{\varepsilon }^{2}\right)p\left(\beta |\omega \right)\\ =\prod_{1}N\left(y_{i}|\sum_{j}x_{\mathrm{ij}}\beta_{j},\sigma_{\varepsilon }^{2}\right)\prod_{j}N\left(\beta_{j}\omega \right) \end{array}$

The choice of prior density, $p\left(\beta |\omega \right)$ , determines whether this posterior mode is equivalent to estimates obtained with the BRR, BL, BEN. In BRR the prior density of marker effects is Gaussian, centered at zero and with common variance, that is, $p\left(\beta_{j}|\sigma_{\beta_{j}}^{2}\right)=N\left(\beta_{j}|{0,\sigma }_{\beta_{j}}^{2}\right)$ where $\sigma_{\beta_{j}}^{2}$ is a prior-variance of marker effects. In BL the prior assigned to marker effects is Double-Exponential (DE) centered at zero and with inverse-scale parameter, $\lambda /\sigma_{\varepsilon }^{2}$ , that is, $p\left(\beta_{j}|\lambda ,\sigma_{\varepsilon }^{2}\right)=\mathrm{DE}\left(\beta_{j}|0,\lambda /\sigma_{\varepsilon }^{2}\right)$ .The prior density of the Bayesian LASSO represents a compromise between the normal and DE densities. Relative to the Gaussian density, the DE places higher mass at zero and have thicker tails, inducing a different type of shrinkage. In particular, relative to BRR, the prior used in the BL induces stronger shrinkage towards zero of estimates of effects of predictors having weak association with the response, and not as much shrinkage of estimates of effects of predictors having strong association with the response. 

### Penalized Semi-Parametric Regression on Marker Effects

An alternative to parametric regressions is to use semi-parametric methods such as reproducing kernel Hilbert spaces (RKHS) regression [56]. A Bayesian RKHS regression for molecular markers regards genetic values as random variables coming from a Gaussian process with a (co)variance structure that is proportional to a kernel matrix **K**, that is, $\mathrm{Cov}\left(g_{i},g_{j}\right)\propto K\left(x_{i},x_{j}\right)$ , where **x**_i_, **x**_j_ are vectors of marker genotypes for the i^th^ and j^th^ individuals, respectively, and *K*(.,.) is a positive definite function evaluated in marker genotypes. One of the advantages of RKHS regression is that it can be used with almost any information set (e.g., covariates, strings, images, graphs). An advantage of RKHS is that the model is represented in terms of n unknowns, which gives RKHS a great computational advantage relative to parametric methods, when p>>n. A disadvantage of the RKHS is that no individual marker effect can be estimated. 

The geostatistical models of Piepho [58] that uses the ridge regression BLUP has also the advantage of having only *n* instead of *p* unknowns when p >> n. The RKHS applied to marker data is related to the spatial models because the ridge regression BLUP corresponds to a spatial model with a quadratic covariance model, while RKHS just replaces this quadratic model with a Gaussian model.

### Examples of the Identification of Marker Effects in Environments for Genomic Selection

#### Do Some (or all) Markers (Genes) have Different (or the same) Response Patterns in Environments? 

Genomic selection is designed to improve complex traits; it focuses mainly on prediction and, as the number of markers increases, prediction of the genomic estimated breeding values (GEBV) is expected to become more accurate, whereas the marker effect is expected to decrease in absolute magnitude. However, parametric regression models also provide the opportunity to examine marker effects and study the possible differential response of markers in environments, that is, the gene × environment interaction effect. In general, the previously examined Bayesian shrinkage methods do not have an associated test for detecting chromosome regions; however, they can be routinely used for examining marker effects in certain chromosome regions.

### Multivariate Analysis of Estimated Marker Effects in Environments

Parametric models such as those described in the previous section yield estimates of marker effects that are environment-specific. In the previous sections, we described how biplots [5, 43, 6] from singular-value decomposition can be used to assess GE. We used these techniques to study GE at the level of estimated marker effects.

Consider a matrix of estimated molecular marker effects, ${\overset{\wedge }{B}}_{p\times q}=\left\lfloor {\overset{\wedge }{\beta }}_{1},..,{\overset{\wedge }{\beta }}_{q}\right\rfloor =\left\{{\overset{\wedge }{\beta }}_{\mathrm{jk}}\right\}$ , whose columns, ${\overset{\wedge }{\beta }}_{k}$ , k = 1,...,q , are estimates of the effects of *p* markers in *q* different environments. The singular value decomposition of this matrix is $\overset{\wedge }{B}=\mathrm{UD}V^{'}$ , where $U_{p\times q}=\left\lfloor \alpha_{1},..,\alpha_{q}\right\rfloor =\left\{\alpha_{\mathrm{jk}}\right\}$ and $V_{q\times q}=\left\lfloor \gamma_{1},..,\gamma_{q}\right\rfloor =\left\{\gamma_{\mathrm{kl}}\right\}$ are ortho-normal matrices that span the row (marker) and column (environment) spaces of $\overset{\wedge }{B}$ , respectively, and **D**_q×q_ is a diagonal matrix whose non-null entries are the singular values of $\overset{\wedge }{B}$ , that is, $D=\mathrm{Diag}\left\{\lambda_{k}\right\}$ . 

The biplot is constructed using the first and second components, that is, **α**_1_, **α**_2_, γ_1_, and γ_2_. Points in the biplot are the marker effects projected in the first two components, and are displayed using the coordinates provided by **α**_1_ and **α**_2_. The “environmental effects” are displayed as vectors whose coordinates are given by **γ**_1_ and **γ**_2_. The length of the vectors approximates the variance accounted for by the specific molecular marker and “environmental effect.” Molecular markers represented in the same direction as the environments had positive effects on those environments, whereas molecular markers located in the opposite direction of the environmental vectors had negative effects on those environments. The cosine of the angle between two environments (or molecular marker effect) approximates the correlation of the two environments (or molecular marker), with an angle of zero indicating a correlation of +1, an angle of 90° (or -90°) a correlation of 0, and an angle of 180° a correlation of -1. 

Biplots of marker effects for several maize datasets comparing maize lines genotyped with several hundred markers and phenotyped in different environments for male and female flowering and some diseases are shown in Crossa *et al*. [32, 33] using the Bayesian shrinkage M-BL model. The maize dataset is from the Drought Tolerant Maize for Africa project of CIMMYT’s Global Maize Program and was obtained by genotyping 300 tropical inbred lines with 1148 SNPs. No pedigree was available for these data.Patterns of Co-Variability of Estimated Marker Effects Across Environments for Maize Flowering Genomic Data.

Traits included here were female flowering (FFL) (or days to silking), male flowering (MFL) (or days to anthesis), as well as the anthesis-silking interval (ASI) evaluated in 300 lines under severe drought stress (SS) and in well-watered (WW) environments [32]. The display of the first two component axes on estimated effects of the SNP markers in the six trait-environment combinations (MFL-SS, MFL-WW, FFL-SS, FFL-WW, ASI-SS, and ASI-WW) obtained from the M-BL model is depicted in Fig. (**7**). The correlation between trait-environment combinations using marker effects and phenotypic data, and the effects of the SNP markers most distant from the center of the biplot are in Tables **4** and **5**, respectively. Table **4** shows the correlations between phenotypic data (upper triangular) and between estimates of marker effects (lower triangular) from the analysis of six trait-environment combinations (ASI-SS, ASI-WW, FFL-SS, FFL-WW, MFL-SS, and MFL-WW) of the maize flowering trial data. Some trait-environment combinations are highly correlated (both phenotypically and genetically). The pattern of correlations between estimated SNP effects reflects the patterns of observed phenotypic correlations [32].

Clearly the two groups of trait-environment combinations are dominated more by the trait (ASI vs FFL and MFL) and less by the environment (SS and WW). Phenotypic outcomes and estimates of marker effects for ASI showed relatively small correlations with those of FFL and MFL; this is because ASI is defined as the difference between FFL and MFL, and these two traits are positively correlated.

Markers with relatively large (in absolute value) estimated effects are identified by name in Fig. (**7**), and their effects are shown in Table **5**. The marker effect on these traits should be interpreted differently than their effect on grain yield, since the favorable marker allele decreases both female and male flowering times, whereas for ASI, the optimal marker should give an ASI of 0. The alleles coded as 1 of SNPs whose estimated effects are located in the left and upper left corner of the biplot (i.e., PZA03551.1, PZA03578.1, PZA03222.1, PZA03385.1, PZB01201.1, and PZB00118.2) increase FFL, MFL, and ASI (they all have positive effects on all trait-environments combinations), whereas those SNPs located on the opposite side of the biplot (lower right corner) (i.e., PZA02587.16, PZA00236.7, PZB0255.1, and PZA00676.2) decrease the value of FFL, MFL, and ASI. Those SNPs whose presence is expected to increase or decrease traits across environments can be viewed as contributing to positive genetic correlations in FFL, MFL, and ASI between environments.

Despite the high heritability (between 0.74 and 0.87) found for flowering time and ASI in this maize trial, results show substantial interaction between molecular marker effects and environment. The biplot in Fig. (**7**) shows SNPs that had very contrasting effects across environments. For example, the minor alleles of SNPs whose estimated effects are located in the upper right corner of the biplot (PZA03592.3, PZB01077.3, and PZB02076.1) increase the anthesis-silking interval under drought and well-watered conditions (Table **5**), but decrease days to male and female flowering. In contrast, the minor alleles of SNPs whose estimated effects are located in the opposite quadrant of the biplot (lower left corner) (PZB00592.1, PHM13183.12, and PZB01964.5) showed a complete rank reversal with respect to the effects of SNPs PZA03592.3, PZB01077.3, and PZB01077.3 on those trait-environment combinations, i.e., a decrease in ASI under SS and WW, and an increase in male and female flowering times. These results are suggestive of important molecular marker effect × environment interaction, which in turn causes genotype × environment interaction. 

### Patterns of Co-Variability of Estimated Marker Effects Across Environments for Exserohilum Turcicum (NCLB) in Maize Genomic Data

**Fig. (8):**
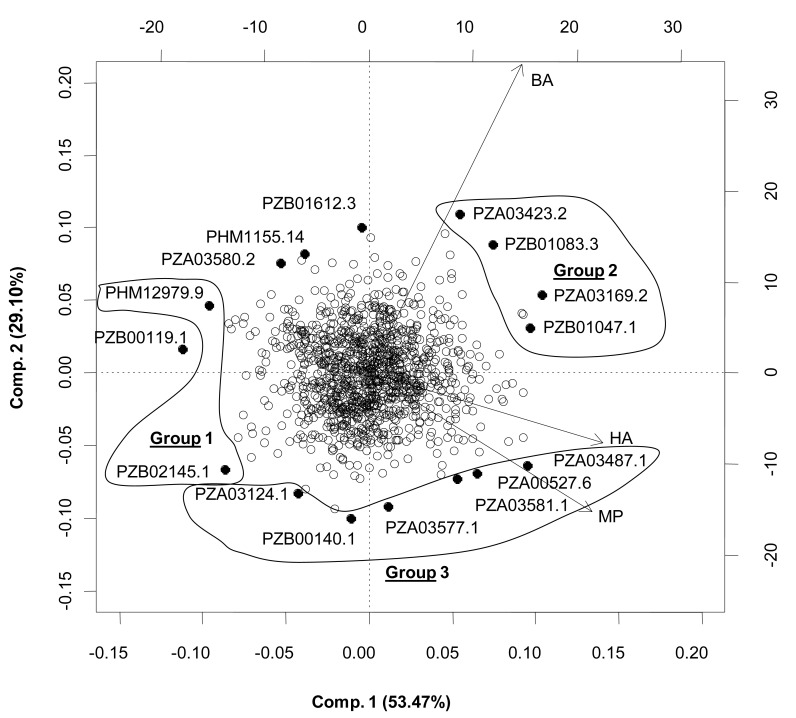
Biplot of the first and second principal component axes (Comp. 1 and Comp. 2) of the Exserohilum turcicum (NCLB) disease effect of 1152 SNPs estimated from the full data M-BL model for the maize dataset in each of three environments: El Batán (México) (BA), Harare (Zimbabwe) (HA), and Mpongwe (Zambia) (MP). Only the effects of 24 SNPs located far from the center of the biplot were identified with the names of their corresponding SNPs. Three groups of environments and molecular markers are delineated as groups 1, 2, and 3 (from Crossa *et al.* [33]).

This section shows marker effects from northern corn leaf blight (NCLB) disease caused by the fungus *Exserohilum turcicum *and evaluated in three international environments: El Batán (Mexico), Harare (Zimbabwe), and Mpongwe (Zambia) [31]. The patterns of marker effects in three environments for *Exserohilum turcicum *(NCLB) in the maize data are depicted in Fig. (**8**). The first two principal components explained 83.58% of the total variability in estimated SNP effects. The correlation among the three environments (based on phenotypic data or on estimates of marker effects) was 0.50 between Mpongwe and Harare and 0.26 between El Batán and Mpongwe and between El Batán and Harare. This pattern of correlations (depicted in Fig. **8**) may indicate the presence of different races of NCLB in these environments, i.e., a pathogen population in El Batán (Mexico) that may be different from populations in Harare and Mpongwe (Southern Africa).

The 16 SNPs with the largest effects (positive or negative) for NCLB are located farthest on the biplot in Fig. (**8**). The presence of the allele coded with 1 of the SNPs in group 1 should confer some degree of resistance to NCLB across environments. The opposite is true for SNPs in groups 2 and 3 (Fig. **8**); here the presence of the allele coded with 1 is expected to favor the disease in El Batán (group 2) and Harare and Mpongwe (group 3). Selection should therefore aim at decreasing this allele. It should be noted that caution must be exercised when interpreting the above results because they are based on one-year data. The prevalence of NCLB races can change from year to year, depending on changes in environmental conditions (e.g., temperature, rainfall, and relative humidity). The variance explained by these 16 SNPs with the largest positive or negative effects ranged between 0.3-1% of the total variance explained by all SNPs. 

## CONCLUDING REMARKS ON THE PLANT GENOMIC STUDIES 

The results of the studies by de los Campos *et al*. [31] and Crossa *et al*. [32, 33] are encouraging; they indicate that models for GS can attain high predictive ability for genetic values of traits of economic interest under contrasting environmental conditions. The results indicate that genomic selection can be used effectively for selecting individuals whose phenotypes for various traits and in various environments have yet to be observed. As the number of available markers increases (as is expected in the near future), larger gains in predictive ability may be attained.

An advantage of models that include a parametric regression on marker covariates, such as M-BL, is that, in addition to estimating genetic values, they also provide information on (estimates of) “marker effects.” This information can be used to attain a better understanding of the genetic architecture of the traits under study and examine the patterns of response of marker effects across environments. In these studies, separate models were fitted to each trait-environment combination. An alternative to these single-environment models for genomic selection is to use multi-environment (or, equivalently, multi-trait) models where genetic values and marker effects on several traits/environments are jointly estimated. Multi-environment models allow borrowing information between correlated environments; thus it can be speculated that multi-environment genomic models can yield similar or even better predictions for individual environments. The literature on genomic selection has focused on single-trait models only; therefore the development of multi-environment and multi-trait models for genomic selection appears to be a natural next step.

Interestlingly, the results of these studies can also be used to generate a better design for field evaluations. For example, they show that prediction of unobserved lines in any of the correlated environments should be relatively accurate, and the scheme for testing these lines in any of those environments should be planned accordingly. It can be speculated that only one of the sets of correlated environments should be included in the trial, because any information lacking for the other environments can be borrowed from the one in use. However, unobserved lines in low correlated environments are expected to be poorly predicted. For example, concerning the analysis of NCLB in the maize data, results showed that estimated marker effects in the two African sites (Mpongwe and Harare) were positively correlated; however, they were negatively correlated with estimates of marker effects obtained for the same trait in Mexico (El Batán), which is suggestive of marker effect × environment interaction. 

The analysis of genetic and genomic data is one of the most challenging and interesting mathematical and statistical problems researchers currently face. Therefore, different models from different areas of mathematical statistical research must be attempted in order to achieve a significant advance in understanding genetic effects. Models that consider complicated systems in biology and are able to study complex gene × gene interactions as well as gene × environment and gene × gene × environment are needed not only for improving prediction of the genetic values of individuals but also for understanding the chromosome regions related to important traits and the main climatic factors affecting these genomic regions. In a recent comprehensive study, Burgueño *et al*. [59] present multi-environment (multi-trait) models for GS and compare the predictive accuracy of these models with: (a) multi-environment analysis without pedigree and marker information, and (b) multi-environment pedigree or/and marker-based models. The authors described a statistical framework for incorporating pedigree and molecular marker information in models for multi-environment data and applied it to data that originated from wheat multi-environment trials. Two prediction problems relevant to plant breeders are considered: predicting the performance of untested genotypes (“newly” developed lines), and predicting the performance of genotypes that have been evaluated in some environments, but not in others. Results confirmed the superiority of models using both marker and pedigree information over those based on pedigree information only. Models with pedigree and/or markers had better predictive accuracy than simple linear mixed models that do not include either of these two sources of information. Burgueño *et al.* [59] concluded that the evaluation of such trials can benefit greatly from using multi-environment GS models.

## Figures and Tables

**Fig. (4):**
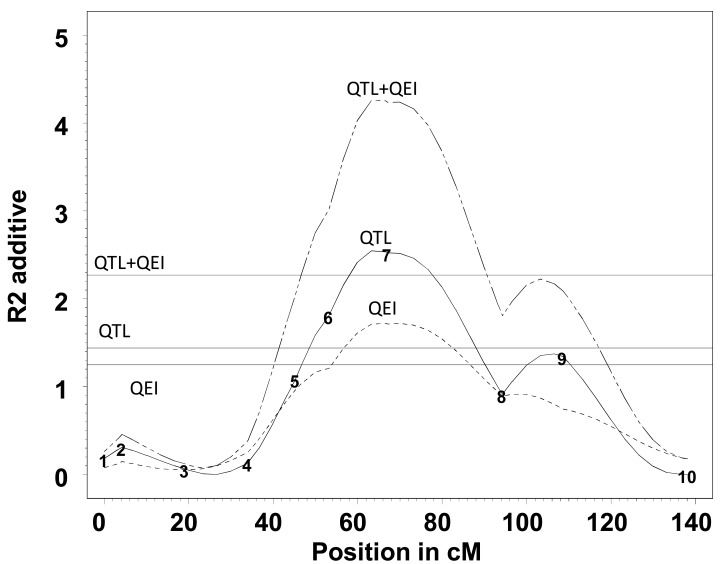
Profile of R^2^ for the additive effects of QTL (solid line), QEI (dotted line), and QTL+QEI (broken line) on grain yield for chromosome 10 (additive). The horizontal lines show the appropriate threshold for the effects QTL+QEI, QTL, and QEI (adapted from Vargas *et al.* [51]).

**Fig. (7):**
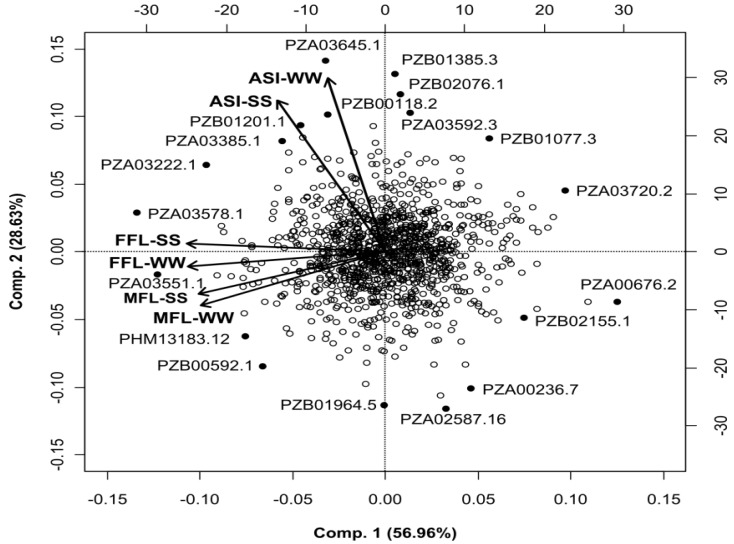
Biplot of the first and second principal component axes (Comp. 1 and Comp. 2) of maize female flowering (FFL) and male flowering (MFL) effects of the 1148 SNPs estimated from the full data model M-BL of the maize dataset in each of two environments, severe water stress (SS) and well watered (WW). A total of six trait-environment combinations (FFL-SS, FFL-WW, MFL-SS, MFL-WW, SS-ASI, and WW-ASI) was formed. Only the effects of the 19 SNPs that are located far from the center of the biplot were identified with their corresponding SNP’s name (filled-in circles) (from Crossa *et al.* [32]).

**Table 1.** Linear Mixed Models Used for Comparing the Prediction of the Missing Genotypes in the Three Maize Trials (M1-MET, M2-MET, and M3-MET). The Overall Mean is µ (Adapted from Burgueño *et al.* [24])

Model	Fixed effects		Random effects			
1	µ	Site	rep(site)		site×genotype	error
2	µ		rep(site)	Site	site×genotype	error
3FA	µ	Site	rep(site)		site(FA)×genotype	error
4FA	µ		rep(site)	Site	site(FA)×genotype	error

**Table 2.** Correlations Between the Predicted and Observed Values of the Missing Genotypes for Three Maize Trials (M1-MET, M2-MET, and M3-MET), Across *Fold* for Four Models (1, 2, 3FA, 4FA). Numbers in Parentheses for Models 2, 3FA, and 4FA Denote the % Change in Correlations with Respect to Model 1 (Adapted from Burgueño *et al.* [24])

Model					
	1	2	3FA		4FA
-------------------M1-MET (overall mean=4.90 Mg ha^-1^)-------------------					
Across *fold*	0.828	0.827 (-0.2)	0.878 (6.0)		0.867 (4.7)
------------------M2-MET (overall mean=5.04 Mg ha^-1^)-------------------					
Across *fold*	0.916	0.916 (0)	0.938 (2.4)		0.938 (2.4)
--------------------M3-MET (overall mean= 5.66 Mg ha^-1^)-----------------					
Across *fold*	0.824	0.824 (0)		0.848 (2.9)	0.852 (3.4)

**Table 3.** Partitioning of Yield Variation at Position 63 cM on Chromosome 10. For Comparison, an Error Estimated from the Median Intra-Block Error was 0.75 (Adapted from Vargas *et al.* [51])

Source of variation	Degrees of freedom	Sum of squares	Mean squares
Environment (E)	7	12777.169	1825.310
G+GE	1680	3212.868	1.914
F2 family (G)	210	1382.102	6.581
GE	1470	1829.700	1.245
Total	1687	15988.970	
----------------------------------------------------------------------------------------------------------------------------------------------------------------			
G+GE	1680	3212.868	1.914
QTL+QEI Chrom. 1-9	---*	1008.879	---
G+GE Chrom. 10 adj.	1680*	2203.988	---
F2 family (G) adj.	210	666.755	3.175
GE adj.	1470	1537.234	1.046
----------------------------------------------------------------------------------------------------------------------------------------------------------------			
G+GE Chrom. 10 adj.	1680*	2203.988	---
QTL+QEI Chrom. 10 63 cM	8	93.868	11.733
QTL main effect	1	56.148	56.148
QEI	7	37.720	5.388
Max.Temp. Flow.	1	8.986	8.986
Residual QEI	6	28.72	4.787
Deviations	1672	2110.121	1.262

* For correction of the grain yield data due to genetic effects on chromosomes 1 through 9, degrees of freedom might be discounted.

**Table 4.** Correlations Between Phenotypic Data (Upper Triangular) and Between Estimates of Marker Effects (Lower Triangular) from the Analysis of Six Trait-Environment Combinations (ASI-SS, ASI-WW, FFL-SS, FFL-WW, MFL-SS, and MFL-WW) of the Maize Flowering Time Trial Data (from Crossa *et al.* [32])

Trait-environment	ASI-SS	FFL-SS	MFL-SS	ASI-WW	FFL-WW	MFL-WW
ASI-SS	---	.446	.109	.728	.315	.109
FFL-SS	.472	---	.926	.221	.700	.633
MFL-SS	.095	.923	---	-.040	.678	.686
ASI-WW	.773	.266	-.037	---	.155	-.123
FFL-WW	.134	.497	.502	.066	---	.948
MFL-WW	-.051	.427	.505	-.173	.971	---

**Table 5.** Biplot of the Principal Component Analysis on the Marker Effects in Each of the Six Trait-Environment Combinations. Estimated Effect of the 19 SNP Molecular Markers Located Farthest from the Center of the Biplot in Fig. (7) from the Maize Flowering Time Trial Data (from Crossa *et al.* [32])

SNP	ASI-SS	FFL-SS	MFL-SS	ASI-WW	FFL-WW	MFL-WW
PZB02155.1	-.01567	-.02808	-.01713	-.01832	-.00957	-.00629
PZA03551.1	.00907	.03973	.02889	.00491	.01938	.01724
PZA03720.2	-.00062	-.02286	-.02511	.00301	-.01575	-.01822
PZA03578.1	.02019	.06300	.04031	.01414	.01310	.01141
PZA03592.3	.02969	-.00158	-.01165	.01705	-.00441	-.00700
PZA03645.1	.02668	.00951	-.00011	.04379	.00257	-.00135
PZA02587.16	-.01695	-.00925	-.00183	-.03975	-.00428	.00134
PZB01385.3	.03359	.00021	-.00960	.02742	-.00337	-.00757
PZA00236.7	-.03476	-.01352	-.00121	-.02162	-.00433	-.00327
PZA00676.2	-.02956	-.05885	-.03312	-.00786	-.01213	-.01068
PZB01077.3	.02016	-.01423	-.02687	.00962	-.00887	-.01099
PHM13183.12	-.00252	.03107	.03278	-.01239	.00734	.01058
PZA03385.1	.02674	.01727	.00896	.02199	.00725	.00306
PZB00592.1	-.01450	.01787	.02865	-.01194	.00966	.01302
PZB01201.1	.02927	.01670	.00614	.02306	.00431	.00128
PZB00118.2	.02900	.01084	.00216	.02443	.00285	-.00150
PZB01964.5	-.02849	.00531	.01561	-.02490	-.00003	.00222
PZA03222.1	.02976	.04733	.02447	.01574	.00902	.00545
PZB02076.1	.02229	-.00645	-.01467	.03059	-.00063	-.00484

## References

[1] Cornelius PL, Crossa J, Seyedsadr M, Kang MS, Gauch HG (1996). Statistical tests and estimators for multiplicative models for cultivar trials. Genotype-by-Environment Interaction.

[2] Crossa J, PL Cornelius (1997). Site regression and shifted multiplicative model clustering of cultivar trial sites under heterogeneity of error variances. Crop Sci.

[3] Gauch HG (1988). Model selection and validation for yield trials with interaction. Biometrics.

[4] Gauch HG, Zobe lRW (1997). Identifying mega-environments and targeting genotypes. Crop Sci.

[5] Gabriel KR (1971). The biplot graphic display of matrices with application to principal component analysis. Biometrika.

[6] Kempton RA (1984). The use of biplots in interpreting variety by environment interactions. J. Agric. Sci.

[7] Gauch HG (2006). Statistical analysis of yield trials by AMMI and GGE. Crop Sci.

[8] Yan W, Tinker NA (2005). An integrated system of biplot analysis for displaying, interpreting, and exploring genotype-by-environment interactions. Crop Sci.

[9] Yan W, Kang MS, Ma B, Woods S, Cornelius PL (2007). GGE biplot vs. AMMI analysis of genotype-by-environment data. Crop Sci.

[10] Gauch HG, Piepho H-P, Annicchiarico P (2008). Statistical analysis of yield trials by AMMI and GGE: Further considerations. Crop Sci.

[11] Yang RC, Crossa J, Cornelius PL, Burgueño J (2009). Biplot analysis of genotype × environment interaction: Proceed with caution. Crop Sci.

[12] Piepho HP (1998). Methods for comparing the yield stability of cropping systems- a review. J. Agronomy Crop Sci.

[13] Piepho HP (2009). Ridge regression and extensions for genome-wide selection in maize. Crop Sci.

[14] Smith A, Cullis BR, Thompson R, Kang MS (2002). Exploring variety-environment data using random effects AMMI models with adjustment for spatial field trends: Part 1: Theory. Quantitative Genetics, Genomics and Plant Breeding.

[15] Smith AB, Cullis BR, Thompson R (2005). The analysis of crop cultivar breeding and evaluation trials: An overview of current mixed model approaches. J. Agric. Sci.

[16] Crossa J, Burgueño J, Cornelius PL, McLaren G, Trethowan R, Krishnamachari A (2006). Modeling genotype × environment interaction using additive genetic covariances of relatives for predicting breeding values of wheat genotypes. Crop Sci.

[17] Piepho HP, Mohring J (2005). Best linear unbiased prediction of cultivar effects for subdivided target regions. Crop Sci.

[18] Burgueño J, Crossa J, Cornelius PL, Trethowan R, McLaren G, Krishnamachari A (2007). Modeling additive × environment and additive × additive × environment using genetic covariances of relatives of wheat genotypes. Crop Sci.

[19] Burgueño J, Crossa J, Cornelius PL, Yang R-C (2008). Using factor analytic models for joining environments and genotypes without crossover genotype × environment interaction. Crop Sci.

[20] Piepho H-P (1997). Analyzing genotype-environment data by mixed models with multiplicative terms. Biometrics.

[21] Piepho H-P (1998). Empirical best linear unbiased prediction in cultivar trials using factor analytic variance-covariance structures. Theor. Appl. Genet.

[22] Oakey H, Verbyla A, Pitchford W, Cullis B, Kuchel H (2006). Joint modeling of additive and non-additive genetic line effects in single field trials. Theor. Appl. Genet.

[23] Cornelius PL, Crossa J (1999). Prediction assessment of shrinkage estimators of multiplicative models for multi-environment cultivar trials. Crop Sci.

[24] Burgueño J, Crossa J, Miguel Cotes J, San Vicente F, Das B (2011). Prediction assessment of linear mixed models for multienvironment trials. Crop Sci.

[25] Fisher RA (1918). The correlation between relatives on the supposition of Mendelian inheritance. Trans. R. Soc. Edinburg.

[26] Malosetti M, Voltas J, Romagoza I, Ullrich SE, van Eeuwjik FA (2004). Mixed models including environmental covariables for studying QTL by environment interaction. Euphytica.

[27] Malosetti M, Boers MP, Bink MCAM, van Eeuwjik FA 2006. Multi-trait QTL analysis based on mixed model with
parsimonious covariance matrices. I: Proceedings of the 8th World
Congress on genetics Applied to Livestock Production, August 13-
18, Belo Horizonte, MG, Brazil. http://www.wcgalp8.org.br/wcgalp8.

[28] Lande R, Thompson R (1990). Efficiency of marker-assisted selection in the improvement of quantitative traits. Genetics.

[29] Meuwissen THE, Hayes BJ, Goddard ME (2001). Prediction of total genetic values using genome-wide dense marker maps. Genetics.

[30] Bernardo R, Yu J (2007). Prospects for genome-wide selection for quantitative traits in maize. Crop Sci.

[31] de los Campos G, Naya H, Gianola D, Crossa J, Legarra A, Manfredi E, Weigel K, Cotes JM (2009). Predicting quantitative traits with regression models for dense molecular markers and pedigree. Genetics.

[32] Crossa J, de los Campos G, Pérez P, Gianola D, Dreisigacker S, Burgueño J, Araus JL, Makumbi D, Yan J, Singh R, Arief V, Banziger M, Braun H-J (2010). Prediction of genetic values of quantitative traits in plant breeding using pedigree and molecular markers. Genetics.

[33] Crossa J, Pérez P, de los Campos G, Mahuku G, Dreisigacker S, Magorokosho C (2011). Genomic selection and prediction in plant breeding. J. Crop Improve.

[34] Pérez P, de los Campos G, Crossa J, Gianola D (2010). Genomic-enabled prediction based on molecular markers and pedigree using the BLR package in R. Plant Genome.

[35] Hayes B, Goddard M (2010). Genome-wide association and genomic selection in animal breeding. Genome.

[36] VanRaden PM (2007). Genomic measures of relationship and inbreeding. Interbull Bulletin.

[37] Yates F, Cochran WG (1938). The analysis of groups of experiments. J. Agric. Sci.

[38] Finlay KW, Wilkinson GN (1963). The analysis of adaptation in a plant breeding programme. Aust. J. Agric. Res.

[39] Eberhart SA, Russell WA (1966). Stability parameter for comparing varieties. Crop Sci.

[40] Williams EJ (1952). The interpretation of interactions in factorial experiments. Biometrika.

[41] Gollob HF (1968). A statistical model which combines features of factor analytic and analysis of variance. Psychometrika.

[42] Mandel J (1969). The partitioning of interaction in analysis of variance. J. Res. Natl. Bur. Stand., Series B.

[43] Gabriel KR (1978). Least squares approximation of matrices by additive and multiplicative models. J. R. Stat. Soc. Series B.

[44] Cornelius PL, Seyedsadr M (1997). Estimation of general linear-bilinear models for two-way tables. J. Stat. Comput. Simul.

[45] Crossa J, Yang R-C, Cornelius PL (2004). Studying crossover genotype × environment interaction using linear-bilinear models and mixed models. J. Agric. Biol. Environ. Stat.

[46] Vargas M, Crossa J, van Eeuwijk FA, Ramirez ME, Sayre K (1999). Using partial least squares, factorial regression and AMMI models for interpreting genotype×environment interaction. Crop Sci.

[47] Van Eeuwijk FA, Malosetti M, Yin X, Struik PC, Stam P (2005). Statistical models for genotype by environment data: from conventional ANOVA models to eco-physiological QTL models. Aus. J. Agric. Res.

[48] Crossa J, Vargas M, van Eeuwijk FA, Jiang C, Edmeades GO, Hoisington D (1999). Interpreting genotype×environment interaction in tropical maize using linked molecular markers and environmental covariables. Theor. Appl. Genet.

[49] Denis J-B (1988). Two-way analysis using covariates. Statistics.

[50] van Eeuwijk FA, Denis J-B, Kang MS, Kang S, Gauch HG (1996). Incorporating additional information on genotypes and environments in models for two-way genotype by environment tables. Genotype-by-environment interaction.

[51] Vargas M, van Eeuwijk FA, Crossa J, Ribaut JM (2006). Mapping QTLs and QTL × environment interaction for CIMMYT maize drought stress program using factorial regression and partial least squares methods. Theor. Appl. Genet.

[52] van Eeuwijk FA, Crossa J, Vargas M, Ribaut JM, Gallais A, Dillmann C, Goldringer I (2000). Variants of factorial regression for analysing QTL by environment interaction. Proceedings of the 11^th^ Meeting of the EUCARPIA Section
Biometrics in Plant Breeding. Quantitative genetics and breeding methods: the way ahead.

[53] Malosetti M, Ribaut JM, Vargas M, Crossa J, van Eeuwijk FA (2008). A multi-trait multi-environment QTL mixed model with application to drought and nitrogen stress trials in maize (*Zea mays* L.). Euphytica.

[54] Tibshirani R (1996). Regression shrinkage and selection *via* the LASSO. J. R. Statist. Soc. B.

[55] Park T, Casella G (2008). The Bayesian LASSO. J. Am. Stat. Assoc.

[56] Gianola D, van Kaam JBCHM (2008). Reproducing kernel Hilbert spaces regression methods for genomic assisted prediction of quantitative traits. Genetics.

[57] Henderson CR (1975). Best linear unbiased estimation and prediction under a selection model. Biometrics.

[58] Piepho HP (2009). Ridge regression and extensions for genome-wide selection in maize. Crop Sci.

[59] Burgueño J, de los Campos G, Weigel K, Crossa J (2012). Genomic prediction of breeding values when modeling genotype × environment interaction using pedigree and dense molecular markers. Crop Sci.

